# The Autonomic Nervous System Regulates the Heart Rate through cAMP-PKA Dependent and Independent Coupled-Clock Pacemaker Cell Mechanisms

**DOI:** 10.3389/fphys.2016.00419

**Published:** 2016-09-27

**Authors:** Joachim Behar, Ambhighainath Ganesan, Jin Zhang, Yael Yaniv

**Affiliations:** ^1^Laboratory of Bioenergetic and Bioelectric Systems, Biomedical Engineering Faculty, Technion-IITHaifa, Israel; ^2^Department of Biomedical Engineering, The Johns Hopkins University of MedicineBaltimore, MD, USA; ^3^Department of Pharmacology, University of California San DiegoSan Diego, CA, USA

**Keywords:** pacemaker cells, mathematical model, autonomic regulation, cAMP-PKA signaling, coupled clock system, phospholamban

## Abstract

Sinoatrial nodal cells (SANCs) generate spontaneous action potentials (APs) that control the cardiac rate. The brain modulates SANC automaticity, via the autonomic nervous system, by stimulating membrane receptors that activate (adrenergic) or inactivate (cholinergic) adenylyl cyclase (AC). However, these opposing afferents are not simply additive. We showed that activation of adrenergic signaling increases AC-cAMP/PKA signaling, which mediates the increase in the SANC AP firing rate (i.e., positive chronotropic modulation). However, there is a limited understanding of the underlying internal pacemaker mechanisms involved in the crosstalk between cholinergic receptors and the decrease in the SANC AP firing rate (i.e., negative chronotropic modulation). We hypothesize that changes in AC-cAMP/PKA activity are crucial for mediating either decrease or increase in the AP firing rate and that the change in rate is due to both internal and membrane mechanisms. In cultured adult rabbit pacemaker cells infected with an adenovirus expressing the FRET sensor AKAR3, PKA activity and AP firing rate were tightly linked in response to either adrenergic receptor stimulation (by isoproterenol, ISO) or cholinergic stimulation (by carbachol, CCh). To identify the main molecular targets that mediate between PKA signaling and pacemaker function, we developed a mechanistic computational model. The model includes a description of autonomic-nervous receptors, post- translation signaling cascades, membrane molecules, and internal pacemaker mechanisms. Yielding results similar to those of the experiments, the model simulations faithfully reproduce the changes in AP firing rate in response to CCh or ISO or a combination of both (i.e., accentuated antagonism). Eliminating AC-cAMP-PKA signaling abolished the core effect of autonomic receptor stimulation on the AP firing rate. Specifically, disabling the phospholamban modulation of the SERCA activity resulted in a significantly reduced effect of CCh and a failure to increase the AP firing rate under ISO stimulation. Directly activating internal pacemaker mechanisms led to a similar extent of changes in the AP firing rate with respect to brain receptor stimulation. Thus, Ca^2+^ and cAMP/PKA-dependent phosphorylation limits the rate and magnitude of chronotropic changes in the spontaneous AP firing rate.

## Introduction

Cardiac diseases affect millions of people each year and their prevalence increases with aging (Docherty, 1990; Kannel and Belanger, 1991). Research has associated a number of cardiac diseases with changes in heart rate (Tsuji et al., 1996; Huikuri and Stein, 2013) and specifically with the failing of pacemaker cell mechanisms such as in sick sinus syndrome and heart failure (reviewed in Yaniv et al., 2013a) or in the presence of mutations in pacemaker cell ion channels (Milanesi et al., 2006; Yaniv and Lakatta, 2013; Baruscotti et al., 2015; Behar and Yaniv, 2016). It is difficult to evaluate the relative financial burden associated directly with the failing of SANC mechanisms, but direct and indirect costs associated with cardiac diseases exceed hundreds of billions $/year. For example, in 2011, the annual costs associated with cardiovascular disease and stroke were $320.1 billion in the US, including $195.6 billion in direct costs and $124.5 billion in indirect costs from lost productivity (Mozaffarian et al., 2015). Thus, there is strong incentive to better understand the etiology of cardiac diseases to better diagnose and design treatments.

**Figure 1:**
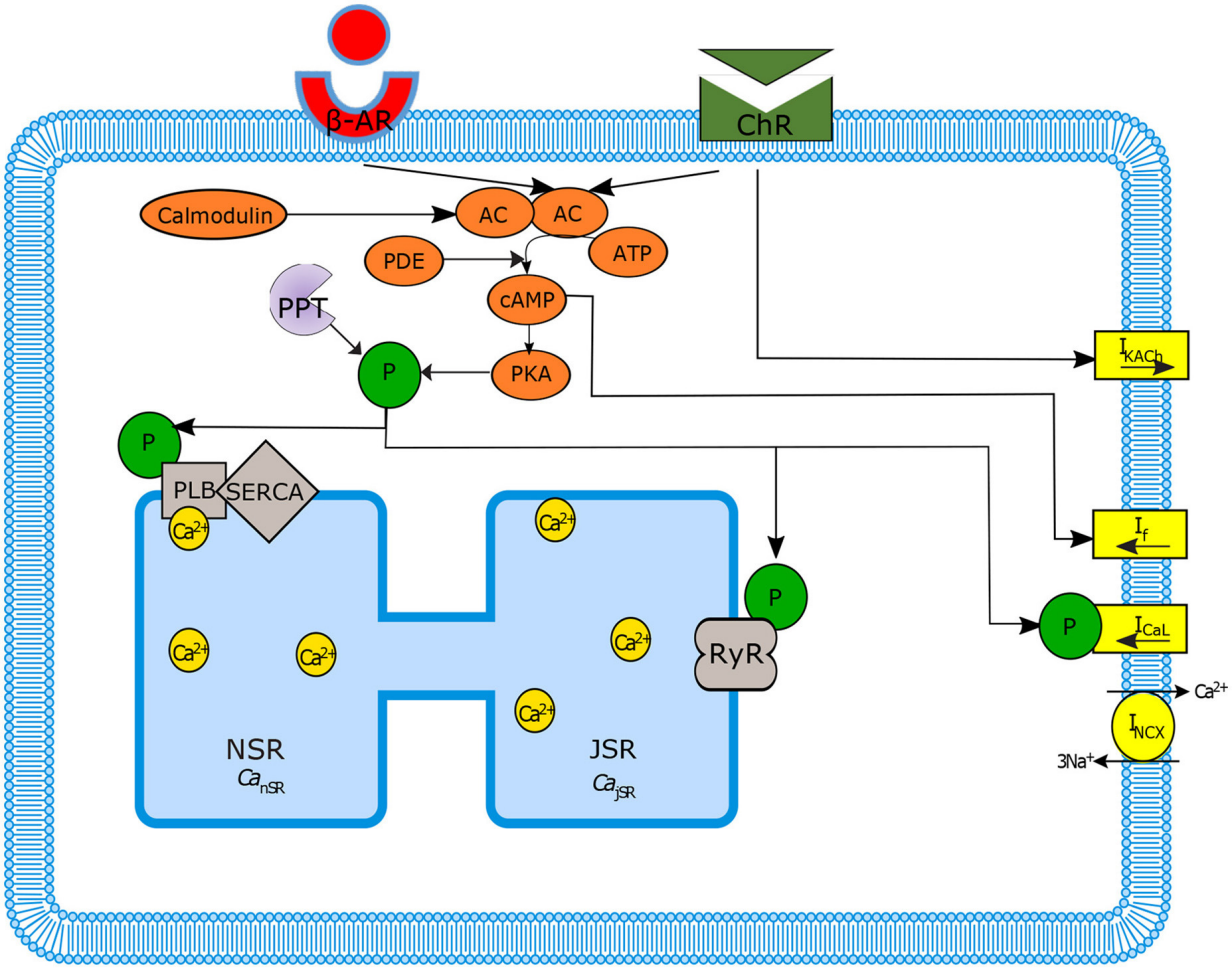
**Schematic diagram of sinoatrial node mechanisms**. Autonomic regulation via adenylyl cyclase-cyclic adenosine monophosphate-Protein kinase A (AC-cAMP-PKA) signaling: the internal pacemaker mechanisms are tightly coupled with cAMP/PKA signaling through the stimulation of G-protein-coupled receptors that activate (adrenergic receptors, β-AR) or inactivate (cholinergic receptors, ChR) AC as well as Ca^2+^-calmodulin activated AC. Only the main ion channels modulated in our model by AC-cAMP-PKA signaling are represented in the figure. SERCA, sarcoplasmic reticulum Ca^2+^ ATPase; PLB, phospholamban; RyR, ryanodine; PDE, phosphodiesterase; PPT, protein phosphatase; P, phosphate.

**Figure 2:**
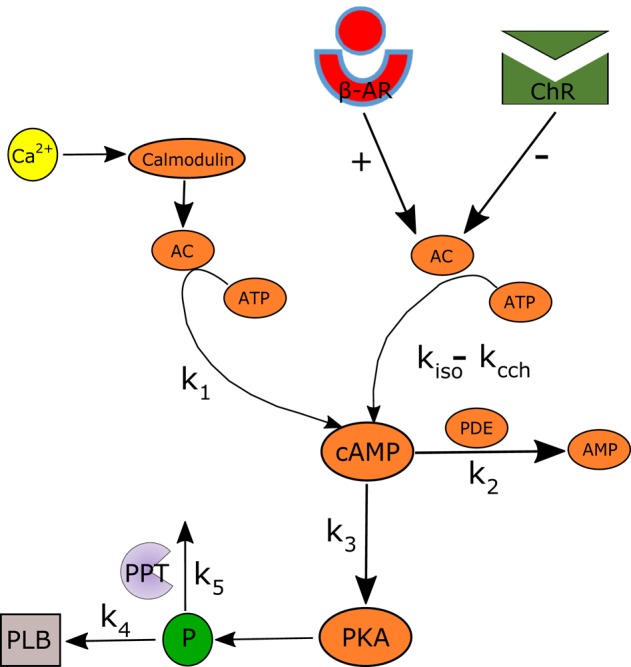
**Schematic illustration of the AC-AC-cAMP-PKA signaling cascade**. Adenylyl cyclase (AC) is activated by adrenergic receptors (β-AR) and calmodulin, and deactivated by cholinergic receptor (ChR) stimulation. Activated AC converts adenosine triphosphate (ATP) into cAMP, which itself is transformed into protein kinase A (PKA). PKA phosphorylates a number of targets, including phospholamban (PLB) proteins, whose phosphorylation level will regulate the activation of SERCA and thus the speed at which Ca^2+^ enters the SR. The model includes two restraining mechanisms that act like brakes: protein phosphatase (PPT), which removes phosphate groups from proteins, and phosphodiesterase (PDE), which breaks the phosphodiester bond in cAMP and degrades its level.

The sinoatrial node function, the heart's primary pacemaker, is regulated by two main signaling cascades: the autonomic nervous system (ANS) and the internal coupled-clock system. In particular dysregulations in the ANS control plays a critical role in coronary artery disease and in the development of ventricular arrhythmias (Schwartz and Priori, 1990; Kjellgren and Gomes, 1993). The control of SANC function by the ANS depends on the balance between the sympathetic and parasympathetic stimulation of G-protein-coupled receptors. In general, adrenergic stimulation (i.e., sympathetic) causes the action potential firing rate to increase and cholinergic stimulation (parasympathetic) causes the action potential firing rate to decrease. However, there is also a non-additive sympathetic-parasympathetic stimulation interaction (Grodner et al., 1970; Levy, 1971). In addition, it has been shown that phasic changes in SANC cycle length occur with respect to the timing, amplitude and duration of the stimulation of the vagus nerve (Jalife and Moe, 1979). Stimulation of G-protein-coupled receptors activate [the β-adrenergic receptor (β-AR)] or inactivate [the cholinergic receptor (ChR)] adenylyl cyclase (AC; Figures 1, 2). The AC generates a high cAMP level, which controls protein kinase A (PKA) activity. Another kind of AC expressed in pacemaker cells is regulated by calmodulin, which is activated by Ca^2+^ cycling. Ca^2+^ cycling is balanced by internal pacemaker mechanisms that are part of the coupled-clock system: interaction between membrane channels, exchangers and pumps (membrane clock, M clock), and internal Ca^2+^ storage (Ca^2+^ clock) (Yaniv et al., 2015b; Figure 1). The internal clock mechanisms interact even without autonomic modulation via a range of node mechanisms: voltage-dependent channels (e.g., via Ca^2+^-dependent inactivation of L-type Ca^2+^ channels; Mangoni et al., 2003), ensembles of local subsarcolemmal Ca^2+^ releases (LCR; Bogdanov et al., 2001), and protein kinase-A (PKA), and calmodulin-dependent kinase II (cAMKII) dependent protein phosphorylation (Trautwein et al., 1987; Takasago et al., 1989; Freeman et al., 1992; Toyofuku et al., 1993). In short, the ensemble of local submembrane Ca^2+^ releases from the sarcoplasmic reticulum (SR) activates the *I*_*NCX*_ exchanger (demonstrating the interaction between the Ca^2+^ and M clocks), the inward current of which has been shown to be one of the main contributors to the diastolic depolarization (DD) (Bogdanov et al., 2006). The M clock also acts on the Ca^2+^ clock through the resetting and refueling of the SR via *I*_*CaL*_ influx (Maltsev and Lakatta, 2009) (Figure 1). Finally, PKA and CaMKII signaling phosphorylate different targets on the M and Ca^2+^ clocks (Figure 1), resulting in changes in the spontaneous action potential (AP) firing rate (Yaniv et al., 2015a) of SANCs. The interaction between these two clocks forms a robust, stable, coupled-clock system that is responsible for SANC automaticity (Yaniv et al., 2015c). Although, there have been many studies of the node mechanisms responsible for the coupling between intrinsic pacemaker molecules of both the M and Ca^2+^ clocks, there is still only a limited understanding of the underlying ionic and molecular mechanisms involved in the crosstalk between the ANS membrane receptors (β-AR or ChR in Figure 1) and the internal mechanisms intrinsic to SANCs. We showed that activation of adrenergic signaling increases AC-cAMP/PKA signaling, which mediates the increase in the SANC AP rate (i.e., positive chronotropic modulation) (Yaniv et al., 2015a). However, there is a limited understanding of the underlying internal pacemaker mechanisms involved in the crosstalk between cholinergic receptors and the decrease in the SANC AP rate (i.e., negative chronotropic modulation). We hypothesize that changes in AC-cAMP/PKA activity are crucial for mediating either decrease or increase in the AP firing rate and that the change in rate is due to both internal and membrane based mechanisms.

In order to gain insights into the many and complex mechanisms involved in heart rate control by both autonomic modulation and internal pacemaker mechanism interaction, we extended an existing computational model of rabbit SANC function. The regulation of the coupled clock by PKA was recently modeled under β-AR stimulation by Yaniv et al. (2015a) The model presented here describes sympathetic and parasympathetic nervous system stimulation of β-AR or ChR and their regulation of AC-cAMP-PKA signaling. In addition, we performed new experiments on spontaneously beating SANCs to assess the role of PKA on AP firing rate modulation in response to ANS stimulation. This work aims to: (a) establish the role of AC-cAMP-PKA signaling as a control mechanism of the crosstalk between autonomic receptors and the coupled-clock system; (b) demonstrate how autonomic modulation allows pacemaker flexibility (i.e., can modulate the AP firing rate) through its modulation of the coupled-clock system; (c) predict the relative role of cAMP/PKA targets on the modulation of the AP firing rate; (d) test if the model can predict the accentuated antagonism effect (i.e., non-additive sympathetic-parasympathetic stimulation interaction); (e) compare the chronotropic effect of directly activating AC-cAMP-PKA to brain receptor stimulation; (f) test how and whether rapid application of CCh starting at different points in the AP cycle affect the AP firing rate. We show for the first time, providing both experimental and theoretical evidence, that the ANS regulates the pacemaker cell function (and thus the resulting AP firing rate) via AC-cAMP-PKA signaling and the muscarinic activated K^+^ current. In addition, the new model predicts that the activation of the SERCA pump via phospholamban (PLB) phosphorylation is a critical player within this regulatory process.

## Methods

### Simulations and experiments

A set of simulations and experiments were performed to assess our hypothesis:

- *In situ*, quantitative, to quantify the relationship between AP firing rate and PKA activity level. We performed experiments on isolated SANCs and measured the spontaneous beating rate (using motion tracking) and imaged PKA activity (using AKAR3 FRET probe) level under different degrees of cholinergic (using ISO) and adrenergic (using CCh) stimulation.
- *In silico*, qualitative, to analyze the effects of sympathetic and parasympathetic stimulation of adrenergic and cholinergic receptors, respectively, on intrinsic coupled clock mechanisms and to understand how the crosstalk is mediated by AC-cAMP-PKA signaling. We performed numerical simulations under basal conditions, and under sympathetic [by isoprenaline (ISO)] and parasympathetic [by carbachol (CCh)] stimulation, and we quantified membrane voltage, main membrane currents, AC-cAMP-PKA signaling, and SR Ca^2+^ fluxes.
- *In silico*, prediction, to identify the dominant pacemaker mechanisms that mediate autonomic regulation crosstalk via AC-cAMP-PKA signaling. We performed numerical simulations where some specific SR or membrane proteins were made PKA phosphorylation insensitive (i.e., their degree of phosphorylation does not change under adrenergic or cholinergic stimulation and is maintained at the basal level), and the model was run for various ISO or CCh concentrations. The output AP firing rates were quantified at steady state.
- *In silico*, qualitative, to compare the dynamics of internal pacemaker mechanisms in response to internal stimulation or autonomic receptor stimulation. We performed numerical simulations to quantify the change in AP firing rate after release of caged cAMP compounds or caged ISO. We also highlighted the changes in membrane voltage, main membrane currents, AC-cAMP-PKA signaling, and SR Ca^2+^ cycling.
- *In silico*, quantitative, to test sympathovagal effects on pacemaker function. We performed numerical simulations to evaluate: (1) the compensatory effects of ChR stimulation by CCh to β-AR stimulation by ISO; (2) the non-additivity effect of ISO and CCh, i.e., that the adrenergic and cholinergic stimulation effects do not sum in a simple additive manner; (3) the phase dependency of the vagal effects. We simulate the dependency of the pacemaker cycle length on the timing of the vagal stimulation during the AP cycle. This is done by simulating flash release of 120 nM of caged CCh at different points in the action potential cycle.

### Experimental protocol

Animals were treated in accordance with the NIH Guide for the Care and Use of Laboratory Animals and the Technion Ethics Committee. The animal protocols have been approved by the Animal Care and Use Committee of the National Institutes of Health (protocol #034LC S2013) and by the Technion (protocol #IL-118-10-13). The rabbits weighed 1.8–2.5 kg and were deeply anesthetized with sodium pentobarbital (50–90 mg/kg) injected to the central ear vein. The adequacy of anesthesia was monitored until ear pinch and jaw tone reflexes were lost. For further details on cell isolation see the Supplementary Material. The PKA activity was imaged and the AP firing rate was measured in SANCs in response to β-AR stimulation (ISO) or ChR stimulation (CCh), as previously described (Yaniv et al., 2015a). To measure PKA activity, adult rabbit pacemaker cells were cultured for 24 h, infected with a 3rd-generation of a genetically encoded A-Kinase activity receptor (AKAR3) FRET probe (Liu et al., 2011), and PKA activity in response to β-AR or ChR stimulations was measured 24 h later. The AP firing rate was measured under the same experimental conditions in another subset of cultured cells. The PKA activity was normalized to the range of PKA activity previously quantified to be between 0.95 (H-89, 10 μM) and 1.4 (IBMX = 100 μM) (Yaniv et al., 2015a). It is known that after 48 h in culture the AP firing rate of SANCs decreases (Yang et al., 2012). One of the main reasons for the decrease in the AP firing rate is the reduced phosphorylation activity in cultured cells (Yaniv et al., 2015a). To bring the cultured cell function as close as possible to basal function, 1 nM of ISO was used to reach a fresh SANC AP firing rate and phosphorylation activity as suggested in Yaniv et al. (2015a). The experimental results under β-AR stimulation (ISO) were reported in previous work (Yaniv et al., 2015a). The experimental results under ChR stimulation (CCh) are reported here for the first time. For further details see Supplementary Material.

### General concept of the numerical model

The activity of a single spontaneously beating rabbit SANC is simulated by our numerical model. The model equations are based on the Maltsev-Lakatta (ML) 2009 model (Maltsev and Lakatta, 2009) and its recent addition of AC-AC-cAMP-PKA signaling under β-AR stimulation by Yaniv et al. (2015a) (denoted as YL). Figure 1 illustrates the extended pacemaker cell numerical model. Figure 1 shows the coupled-clock system concept: the different compartments of the cells (cytosol, submembrane, and SR), the major ion channels (yellow rectangles) that constitute the M Clock, and the proteins on the SR (gray shapes) that constitute the Ca^2+^ clock. Figure 1 also illustrates the AC-cAMP-PKA signaling: the internal pacemaker mechanisms are tightly coupled with AC-cAMP-PKA signaling through the stimulation of G protein-coupled receptors (β-AR or ChR, see Figure 1) that activate (adrenergic) or inactivate (cholinergic) AC. In pacemaker cells, Ca^2+^ -activated ACs also exist (Mattick et al., 2007). The AC transforms adenosine triphosphate (ATP) into cAMP, which activates PKA. The model takes into account the phosphorylation of key ion channel proteins on the membrane (*I*_*CaL*_, *I*_*f*_) and on the SR (PLB and RyR) (Trautwein et al., 1987; Takasago et al., 1989; Freeman et al., 1992; Toyofuku et al., 1993). The ChR stimulation also opens muscarinic K^+^ current channels (*I*_*KACh*_) (Trautwein and Dudel, 1958). The model includes two restraining mechanisms that act like brakes: protein phosphatase (PPT), which removes phosphate groups from proteins, and phosphodiesterase (PDE), which breaks the phosphodiester bond in cAMP and degrades its level (see Figures 1, 2). When experimental data exist (see description of each equation below), low-order non-linearity was used to fit the prediction of the numerical model to the experimental data.

### Membrane potential

The following paragraphs expand on some specific parts of the numerical model. A full description of the model is provided in the online supplement. It is assumed that the net current passing through the membrane is determined by the following components:

(1) $$\begin{array}{lll} I & = & I_{CaL}+I_{CaT}+I_{bCa}+I_{f}+I_{st}+I_{Kr}+I_{Ks}+I_{NaK}+I_{NCX}+\\ I_{bNa}+I_{to}+I_{sus}+I_{KACh}, \end{array}$$

where *I* is the sum of all membrane currents, *I*_*CaL*_ the L- type Ca^2+^ current, *I*_*CaT*_ the T-type Ca^2+^ current, *I*_*bCa*_ the Ca^2+^ background current, *I*_*f*_ the hyperpolarization- activated funny current, *I*_*st*_ the sustained inward current, *I*_*Kr*_ the rapidly activating delayed rectifier K^+^ current, *I*_*Ks*_ the slow activating delayed rectifier K^+^ current, *I*_*NaK*_ the Na^+^-K^+^ pump current, *I*_*NCX*_ the Na^+^-Ca^2+^ exchanger current, *I*_*bNa*_ the Na^+^ dependent, *I*_*to*_ and *I*_*sus*_ the 4-aminopyridine- sensitive currents, and *I*_*KACh*_ the ACh activated muscarinic current. The formulation of the ACh-activated muscarinic current, *I*_*KACh*_, is adapted from Demir et al. (1999), with a maximal conductance taken to be *g*_*KACh*_ = 0.3133 nS∕pF. Of note, *I*_*KACh*_, is directly activated by the concentration of ACh and not by the cAMP concentration or the PKA activity level (see Figure 1). The rate of change of the membrane potential can be obtained using the following equation:

(2) $$\begin{array}{l} \frac{dV_{m}}{dt}=-\left(\frac{1}{C_{m}}\right)\times I, \end{array}$$

where *C*_*m*_ is the membrane capacitance and *V*_*m*_ is the membrane potential.

### Numerical integration

Software was developed in MATLAB (The MathWorks, Inc., Natick, MA, US). Numerical integration was performed using the ode15s stiff solver. To ensure that steady state was reached, the model was run for 900 s before reporting any results.

### AC-cAMP-PKA signaling

Figure 2 illustrates the main cAMP production and degradation mechanisms that are included in the numerical model. The ATP is transformed into cAMP by activated AC. It is assumed that the total AC activation is modulated by β-AR stimulation (rate constant *k*_*iso*_ = *F*([*ISO*])) and ChR stimulation (*k*_*cch*_ = *F*([*CCh*])) as well as by calmodulin (*k*_1_ = *F*([*f*_*CMi*_])). The cAMP is converted into PKA following a transformation whose kinetic is modulated by *k*_3_ = *F*([*cAMP*]). The phosphodiester bond in cAMP is broken by PDE following a kinetic with rate constant *k*_2_ = *F(cAMP)*. Finally, PLB is phosphorylated by PKA following a kinetic value controlled by *k*_4_ = *F*([*PKA*]) and degraded by PPT (*k*_5_ = *F*([*PLB*_*p*_])). *k*_4_ and *k*_5_ were obtained by curve fitting, using the experimental data from Vinogradova et al. (2006, 2008). For simplicity, only first order kinetics are considered. The equations modeling the cAMP and PLB_*p*_ dynamics and following the model shown in Figure 2 are:

(3) $$\begin{array}{lll} \frac{d\left[AC\right]}{dt} & = & \frac{d{\left[AC\right]}_{Ca^{2+}}}{dt}+\frac{d{\left[AC\right]}_{GPCR}}{dt} \end{array}$$

(4) $$\begin{array}{lll} \frac{d\left[AC\right]}{dt} & = & k_{1}\cdot \left[ATP\right]+\frac{dGa}{dt}+\frac{dGi}{dt} \end{array}$$

(5) $$\begin{array}{lll} \frac{d\left[AC\right]}{dt} & = & k_{1}\cdot \left[ATP\right]+\left(k_{iso}-k_{cch}\right)\cdot \left[ATP\right] \end{array}$$

(6) $$\begin{array}{lll} \frac{d\left[cAMP\right]}{dt} & = & \frac{d\left[AC\right]}{dt}-k_{2}\cdot \left[cAMP\right]-k_{3}\cdot \left[cAMP\right] \end{array}$$

(7) $$\begin{array}{l} \frac{d\left[cAMP\right]}{dt}=\left(k_{iso}-k_{cch}\right)\cdot \left[ATP\right]+k_{1}\cdot \left[ATP\right]\\ -k_{2}\cdot \left[cAMP\right]-\;k_{3}\cdot \left[cAMP\right] \end{array}$$

(8) $$\begin{array}{lll} \frac{d\left[PLB_{p}\right]}{dt} & = & k_{4}\cdot \left[PKA\right]-k_{5}\cdot \left[PLB_{p}\right], \end{array}$$

with,

(9) $$\begin{array}{lll} k_{iso} & = & 0.0070+0.1181\cdot \frac{{\left[ISO\right]}^{0.8664}}{48.1212^{0.8664}+{\left[ISO\right]}^{0.8664},} \end{array}$$

(10) $$\begin{array}{lll} k_{cch} & = & 0.0146\cdot \frac{{\left[CCh\right]}^{1.4402}}{51.7331^{1.4402}+{\left[CCh\right]}^{1.4402}}. \end{array}$$

**Figure 3:**
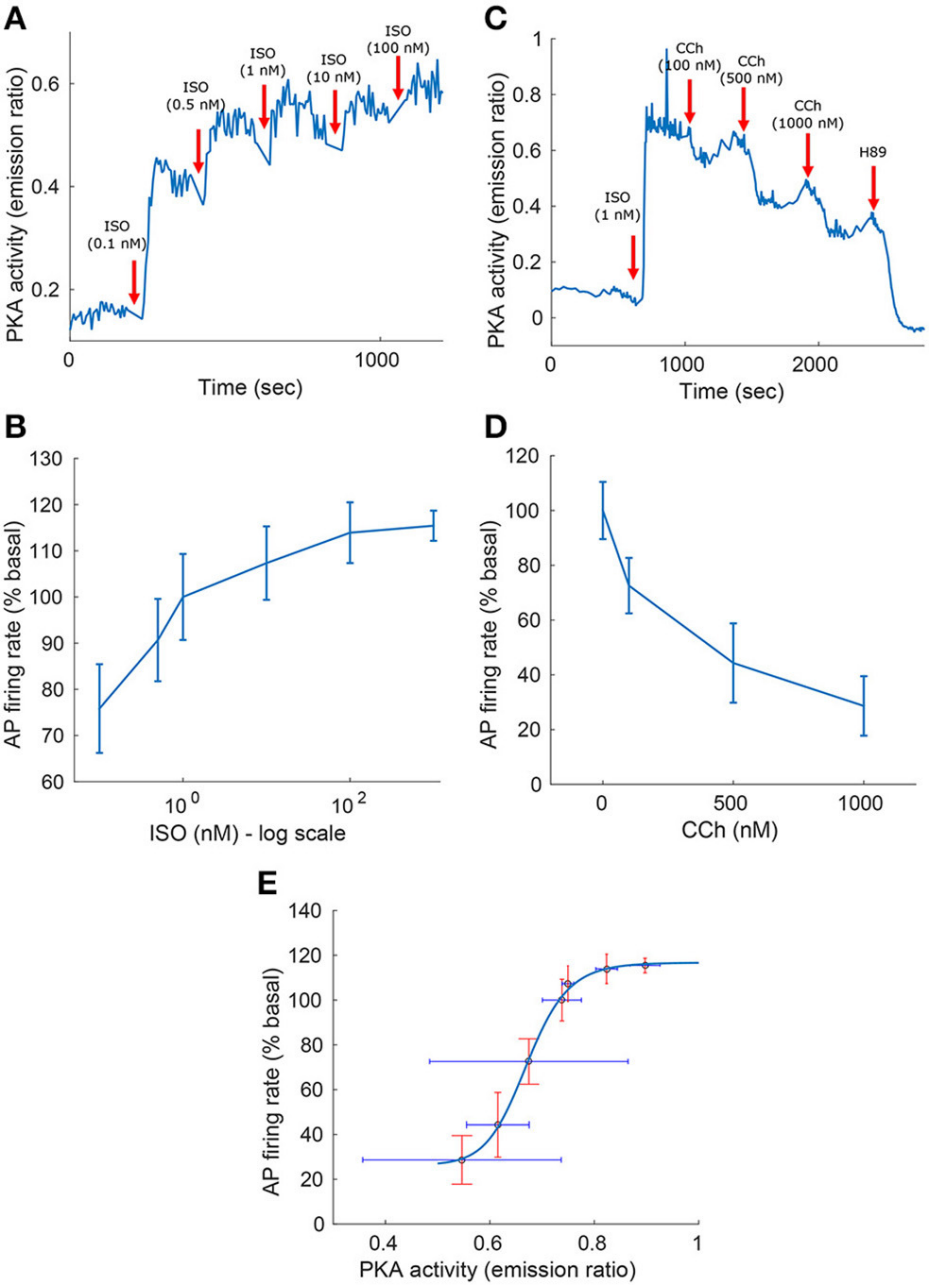
**Experimental measurements of PKA activity and action potential (AP) firing rate as a function of sympathetic stimulation (via adrenergic receptors, A,B) using isoproterenol (ISO) and parasympathetic stimulation (via cholinergic receptors, C,D) using carbachol (CCh) in rabbit pacemaker cell. (A,C)** Show representative examples, **(B,D)** present average data for *n* = 5 and *n* = 4 pacemaker cells (from 5 and 4 rabbits, respectively), respectively, with the vertical bars representing the standard errors. **(E)** Shows the relationship between the AP firing rate and PKA activity, as obtained from the ISO and CCh measurements. Equation for the curve fitting of the AP-PKA relationship: 26.4434+90.3024·*x*^16.9029^/(0.6713^16.9029^+*x*^16.9029^).

Equation (3) models the AC activated by internal *Ca*^2+^ (denoted $\;{\left[AC\right]}_{Ca^{2+}}$) or by GPCR (denoted [*AC*]_*GPCR*_), which sense the nervous stimulations outside the cell and activate intracellular AC. Equation (4) breaks down the GPCR activation of the primary effector protein AC into its two components: positive activation caused by the stimulation of adrenergic receptors and inactivation caused by the stimulation of cholinergic receptors. Equation (5) expresses the positive activation and inactivation as a function of ISO, CCh, and the level of ATP. Finally, Equations (6, 7) give the rate of cAMP change as a function of the ATP that is converted into cAMP via Ca^2+^-AC or via GPCR, and the cAMP that is degraded through the production of PKA or by PDE. Brackets represent the concentration of a substance (e.g., [cAMP], [ATP]) or the level of activation of a substance (e.g., [*PKA*] ∈ [0 − 1], [*PLB*_*p*_] ∈ [0 − 1]). The terms for *k*_1_ − *k*_5_ are adapted from the YL model (Yaniv et al., 2013d, 2015a). The functions *k*_*iso*_ and *k*_*cch*_ are evaluated using the experimental relationships between the AP firing rate and cAMP (AP-cAMP) and cAMP and ATP (cAMP-ATP) (Yaniv et al., 2015a). In addition, to estimate *k*_*iso*_, experimental measurements from Yaniv et al. (2015a) (republished here in Figures 3A,B) are used to evaluate the relationship between the AP firing rate and the ISO level (AP-ISO). For *k*_*cch*_ we used our new experimental measurements to evaluate the AP-CCh relationship (see Figures 3C,D). Because the data for the ISO and CCh experiments were obtained on cultured cells, which typically have a reduced basal AP firing rate with respect to fresh cells (Yang et al., 2012), the basal AP firing rate is assumed to be obtained for ISO = 1*nM* (13). The relationship between cAMP and PKA was adapted from the Saucerman et al. model (Saucerman et al., 2003). More details are available in the Supplementary Material.

### Ion channel activation by cAMP/PKA

The L-type channel current is modulated by the level of PKA-dependent phosphorylation (Trautwein et al., 1987). For our model we assume a maximal activation of +80% of *I*_*CaL*_ in response to β-AR stimulation (Vinogradova et al., 2002) and a maximal inactivation by CCh of −20% (Lyashkov et al., 2009). The funny current, *I*_*f*_, has been shown to be modulated directly by cAMP binding to the channel (Difrancesco and Tortora, 1991). The formulation of the cAMP mediation of *I*_*f*_ was adapted from the YL model (Yaniv et al., 2015a).

### Activation of SERCA by PKA

The SERCA pump is assumed to depend on phosphorylated PLB_*p*_, following the equation:

(11) $$\begin{array}{l} j_{up}=P_{up}\cdot F\left(\left[PLB_{p}\right]\right)\cdot \frac{\left[Ca_{i}\right]}{\left(\left[Ca_{i}\right]+K_{up}\right)}, \end{array}$$

where *F*([*PLB*_*p*_]) describes the modulation of *j*_*up*_ by phosphorylated PLB, [*Ca*_*i*_] is the Ca^2+^ cytosolic concentration, and *P*_*up*_ and *K*_*up*_ are model constants. The analytical expression of *F*([*PLB*_*p*_]) is:

For [*PLB*_*p*_] ≤ 0.23,

(12) $$\begin{array}{l} F\left(\left[PLB_{p}\right]\right)=1.698\cdot \frac{{\left[PLB_{p}\right]}^{13.584}}{0.2240^{13.584}+{\left[PLB_{p}\right]}^{13.584}}, \end{array}$$

and for [*PLB*_*p*_] > 0.23,

(13) $$\begin{array}{l} F\left(\left[PLB_{p}\right]\right)=3.3931\cdot \frac{{\left[PLB_{p}\right]}^{4.0695}}{0.2805^{4.0695}+{\left[PLB_{p}\right]}^{4.0695}}-0.0952. \end{array}$$

The function *F*([*PLB*_*p*_]), which quantifies the modulation of the SERCA by PLB_*p*_, is phenomenological. It was assumed to be equal to 0.23 in the basal state (i.e., [*PLB*_*p*_] = 0.23), and to increase or to decrease as the degree of phosphorylation of PLB changes.

### Caged cAMP/ISO experiment

Caged compounds are light-sensitive probes that functionally encapsulate biomolecules in an inactive form. Irradiation will liberate the caged molecules and allow the perturbation of biological processes (Ellis-Davies, 2007). Assuming first-order kinetics, the photo-release of a caged cAMP compound is described by the equation:

(14) $$\begin{array}{l} \frac{df_{cb}}{dt}=k_{cAMP,on}\cdot \left[cAMP\right]\cdot \left(1-f_{cb}\right)-k_{cAMP,off}\cdot f_{cb,} \end{array}$$

and after flash photolysis induction is described by:

(15) $$\begin{array}{l} \frac{df_{cb}}{dt}=k_{cAMP,on}\cdot \left[cAMP\right]\cdot \left(1-f_{cb}\right), \end{array}$$

where *f*_*cb*_ ∈ [0 1] corresponds to the ratio of caged to uncaged cAMP, *k*_*cAMP, on*_ is the rate of cAMP caging and *k*_*cAMP, off*_ the rate of cAMP release from the cage. We assumed *C*_*b*_ = 50 μM of caged cAMP, similar to Tanaka et al. (1996), and a dissociation constant of *k*_*cAMP, off*_ = 0.3 ms^−1^ (Ellis-Davies, 2007) (data reported for Invitrogen DMNPE-cAMP). *k*_*cAMP, on*_ was assumed to be very small (10^−5^ mM^−1^ ms^−1^), as no experimental data were found for this constant. The equation modeling the cAMP rate is modified to include the changes in cAMP dynamics during photolysis of the caged cAMP:

(16) $$\begin{array}{l} \frac{d\left[cAMP\right]}{dt}=\frac{\left(\left(k_{iso}-k_{cch}\right)\cdot \left[ATP\right]+k_{1}\cdot \left[ATP\right]-k_{2}\cdot \left[cAMP\right]-k_{3}\cdot \left[cAMP\right]\right)}{6000}-C_{b}\cdot E_{M,p}\cdot \frac{df_{cb}}{dt}, \end{array}$$

where *E*_*M, p*_ is a constant used to convert cAMP from mM to pmol/mg protein. The division by 6000 converts the units from min^−1^ to ms^−1^.

For the caged ISO experiment, a similar modeling was used. During flash photolysis:

(17) $$\begin{array}{l} \frac{df_{ci}}{dt}=k_{ISO,on}\cdot \left[ISO\right]\cdot \left(1-f_{ci}\right)-k_{ISO,off}\cdot f_{ci}, \end{array}$$

and after flash photolysis:

(18) $$\begin{array}{l} \frac{df_{ci}}{dt}=k_{ISO,on}\cdot \left[ISO\right]\cdot \left(1-f_{ci}\right), \end{array}$$

where *f*_*ci*_ ∈ [0 1] corresponds to the ratio of caged to uncaged cAMP, *k*_*ISO, on*_ is the rate of ISO caging, and *k*_*ISO, off*_ is the rate of ISO release from the cage. We assumed *C*_*i*_ = 3 μM of caged ISO, similar to Tanaka et al. (1996), and a dissociation constant of *k*_*ISO, off*_ = 0.11 ms^−1^ (Muralidharan and Nerbonne, 1995). *k*_*ISO, on*_ was assumed to be very small (10^−7^ mM^−1^ ms^−1^), as no experimental data were found for this constant. The rate change in ISO concentration can be described by:

(19) $$\begin{array}{l} \frac{d\left[ISO\right]}{dt}=-\;C_{i}\cdot \frac{df_{ci}}{dt}. \end{array}$$

## Results

To test the model predictions, we experimentally quantified the relationship between the AP firing rate and PKA activity level in response to β-AR or ChR stimulation. Figure 3 shows the experimental measurements of PKA activity and AP firing rate in response to sympathetic stimulation (via β-AR stimulation, Figures 3A,B) using ISO, or parasympathetic stimulation (via ChR receptors, Figures 3C,D) using CCh. In the basal state (after 1 nM of ISO), the AP firing rate was 183 bpm, which falls in the physiological range for fresh cells (Lyashkov et al., 2007), and the PKA activity (emission ratio) was 0.74. The high basal level of PKA activity in SANC is not surprising due to high basal cAMP level of isolated rabbit SANC (Vinogradova et al., 2006). Combining the experimental measurements from Figures 3A–D allowed us to evaluate the relationship between the AP firing rate and the PKA activity level in the entire physiological range (Figure 3E). This exhibits a sigmoid-like trend: linear around the basal PKA activity level and then rapidly saturating as the PKA activity level is increased or decreased. The measured AP firing rate varied between 74 and 122% (% basal) and the PKA activity level between 0.72 and 0.93 (emission ratio).

**Figure 4:**
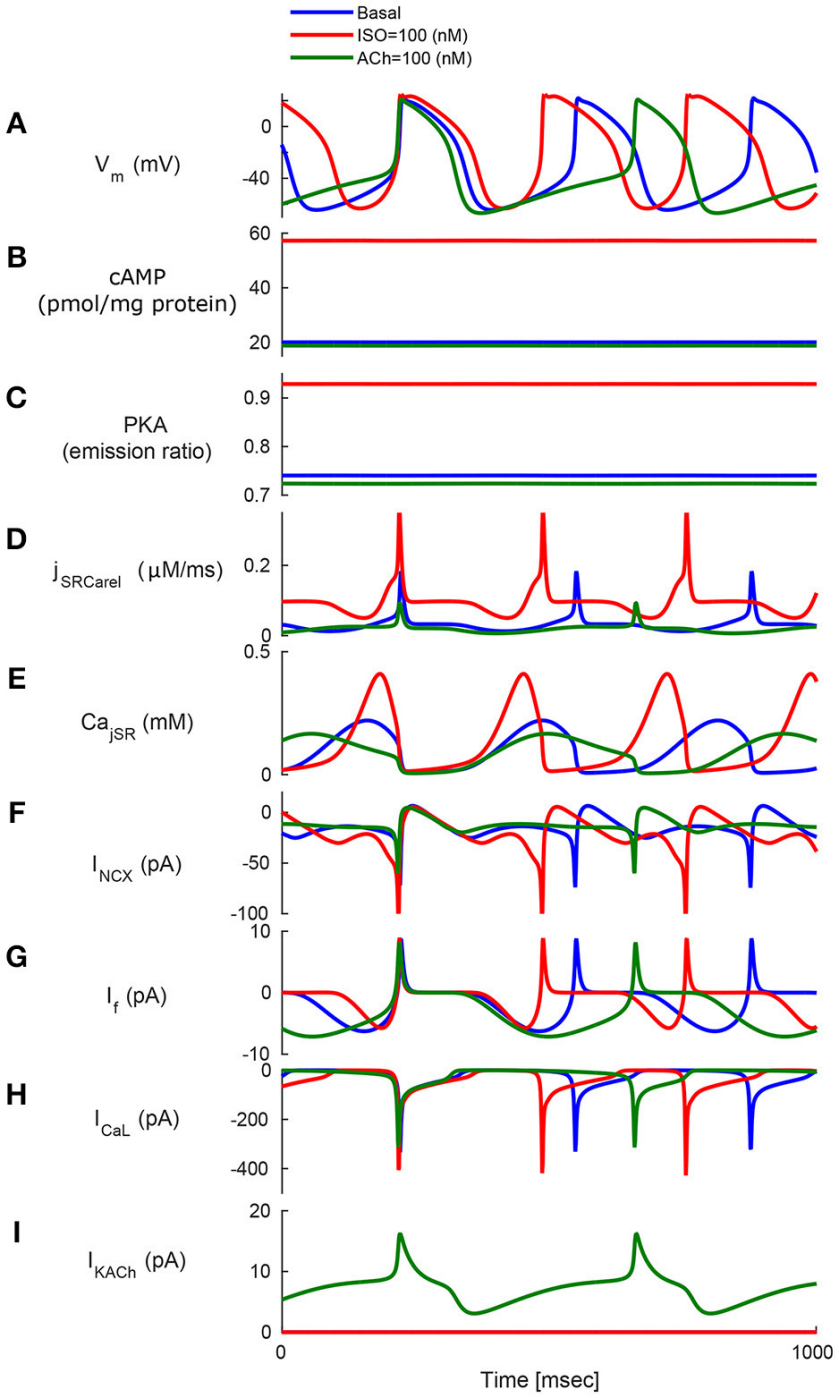
**Model simulations in response to sympathetic and parasympathetic stimulation**. Membrane voltage, main membrane currents, AC-cAMP-PKA signaling, and Ca^2+^ cycling (flux and concentration) in the sarcoplasmic reticulum (SR) in the basal state or in response to sympathetic stimulation (via adrenergic receptors, red curve) using isoproterenol (ISO) or parasympathetic stimulation (via cholinergic receptors, green curve) using carbachol (CCh). The panels show: **(A)** The membrane voltage (V_*m*_); **(B)** cAMP level; **(C)** PKA activity level; **(D)** the flux of Ca^2+^ exiting the sarcoplasmic reticulum (*j*_*SRCarel*_); **(E)** Ca^2+^ concentration in the junctional sarcoplasmic reticulum compartment (Ca_*jSR*_); **(F)** the Na^+^-Ca^2+^ exchanger current (*I*_*NCX*_); **(G)** funny-current (*I*_*f*_); **(H)** L-type current (*I*_*CaL*_); and **(I)** muscarinic activated current (*I*_*KACh*_).

**Figure 5:**
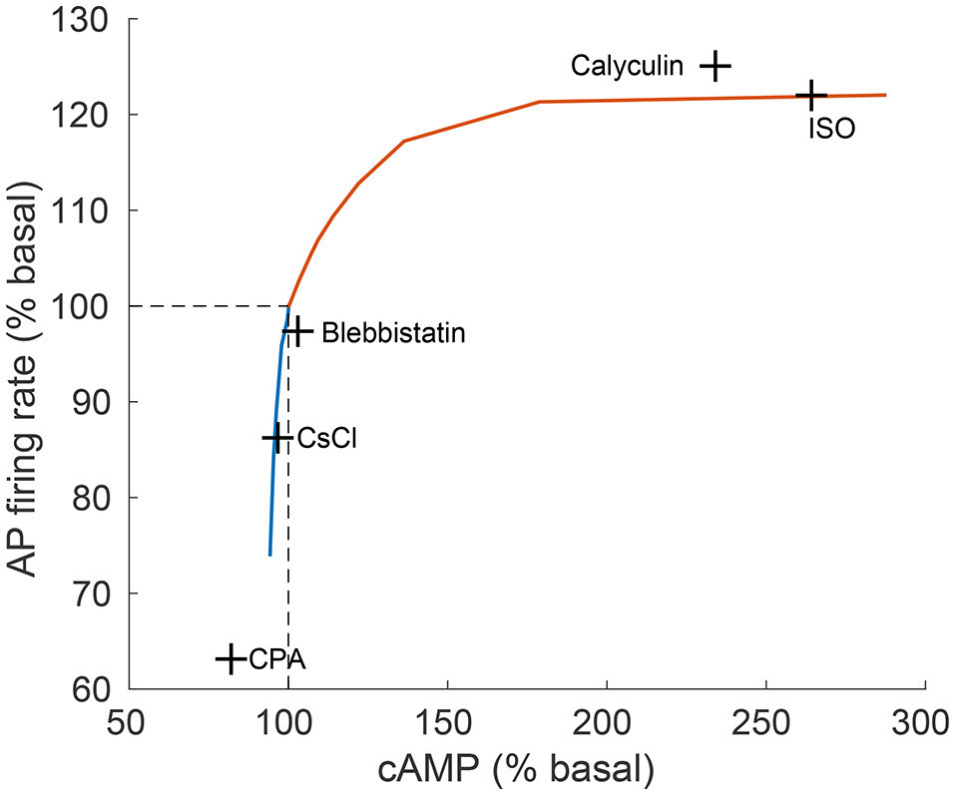
**Relationship between action potential (AP) firing rate and cAMP level**. The relationship between action potential (AP) firing rate and cAMP, as predicted by the model (continuous line) in comparison to experimental results (crosses) from Yaniv et al. (2013d). The model predictions are obtained for varying quantities of ISO (orange curve) and CCh (blue curve). The concentrations of drugs used for the experiments were: isoproterenol (ISO, 1 μM), calyculin A (1 μM), blebbistatin (10 μM), caesium chloride (CsCl, 2 mM), cyclopiazonic acid (CPA, 5 μM; Yaniv et al., 2011).

To test whether AC-cAMP-PKA signaling crosstalk autonomic activity to SANC function, we performed numerical simulations under basal conditions, and under sympathetic or parasympathetic stimulation, and we quantified AC-cAMP-PKA signaling, membrane voltage, main membrane currents, and SR Ca^2+^ fluxes. Figure 4 shows the model simulation under basal conditions (i.e., without adrenergic or cholinergic stimulation, in blue), under adrenergic stimulation (in red), or under cholinergic stimulation (in green). The action potential firing rate increases under adrenergic stimulation and decreases under cholinergic stimulation (Figure 4A). Sympathetic stimulation by ISO leads to increased PKA and cAMP levels (Figures 4B,C), which results in modulated Ca^2+^ cycling within the cell, and in particular the amount of Ca^2+^ that is ejected via the ryanodine (RyR) channels (Figures 4D,E), which in turn stimulates the *I*_*NCX*_ (Figure 4F). A parallel increase in cytoplasmic Ca^2+^ further activates AC-cAMP-PKA signaling via calmodulin activated AC (see Figure 1). Additionally, cAMP signaling causes a voltage shift in the *I*_*f*_ activation curve, which leads to an increased contribution of *I*_*f*_ to early diastolic depolarization (Figure 4G). The L-type channel current, *I*_*CaL*_, is activated by the increase in PKA activity level under ISO (Figure 4H). Change in coupled-clock mechanisms lead to faster initiation of early DD and thus a higher AP firing rate (Figure 4A). In the case of parasympathetic stimulation of the ChR receptors, the PKA, and cAMP levels were lowered (Figures 4B,C). Ca^2+^ that is ejected via the RyR channels is lowered (Figure 4D), and as a consequence the activation of *I*_*NCX*_ is lowered (Figure 4F). *I*_*CaL*_ is inactivated by the decrease in PKA. In addition, the muscarinic current, *I*_*KACh*_, is activated and contributes to the early DD together with *I*_*f*_ and *I*_*NCX*_ (Figure 4I). The changes in *I*_*NCX*_, *I*_*KACh*_, and *I*_*f*_ result in a slower initiation of early DD and thus a lower AP firing rate. In the basal state, the AP firing rate was 183 bpm, which falls in the physiological range for fresh SANCs (Lyashkov et al., 2007). In the case of [ISO] = 100 nM, the PKA activity level increased by 26% and the cAMP level by 186% with respect to the basal level. In response to ChR (100 nM), the PKA activity level decreased by 2% and the cAMP level by 6%. The resulting AP firing rate was 223 bpm for [ISO] = 100 nM (i.e., an increase of 22% with respect to the basal level) whereas the AP firing rate was 135 bpm for CCh = 100 nM (i.e., a reduction of 26% with respect to the basal level). Of note, *I*_*KACh*_ was only present in the case of parasympathetic stimulation, as expected. When running the model for a large range of β-AR (0–100 nM) and ChR (0–100 nM), the AP firing rate-cAMP relationship is coherent with experimental data published in previous work (Yaniv et al., 2013d; see Figure 5).

**Figure 6:**
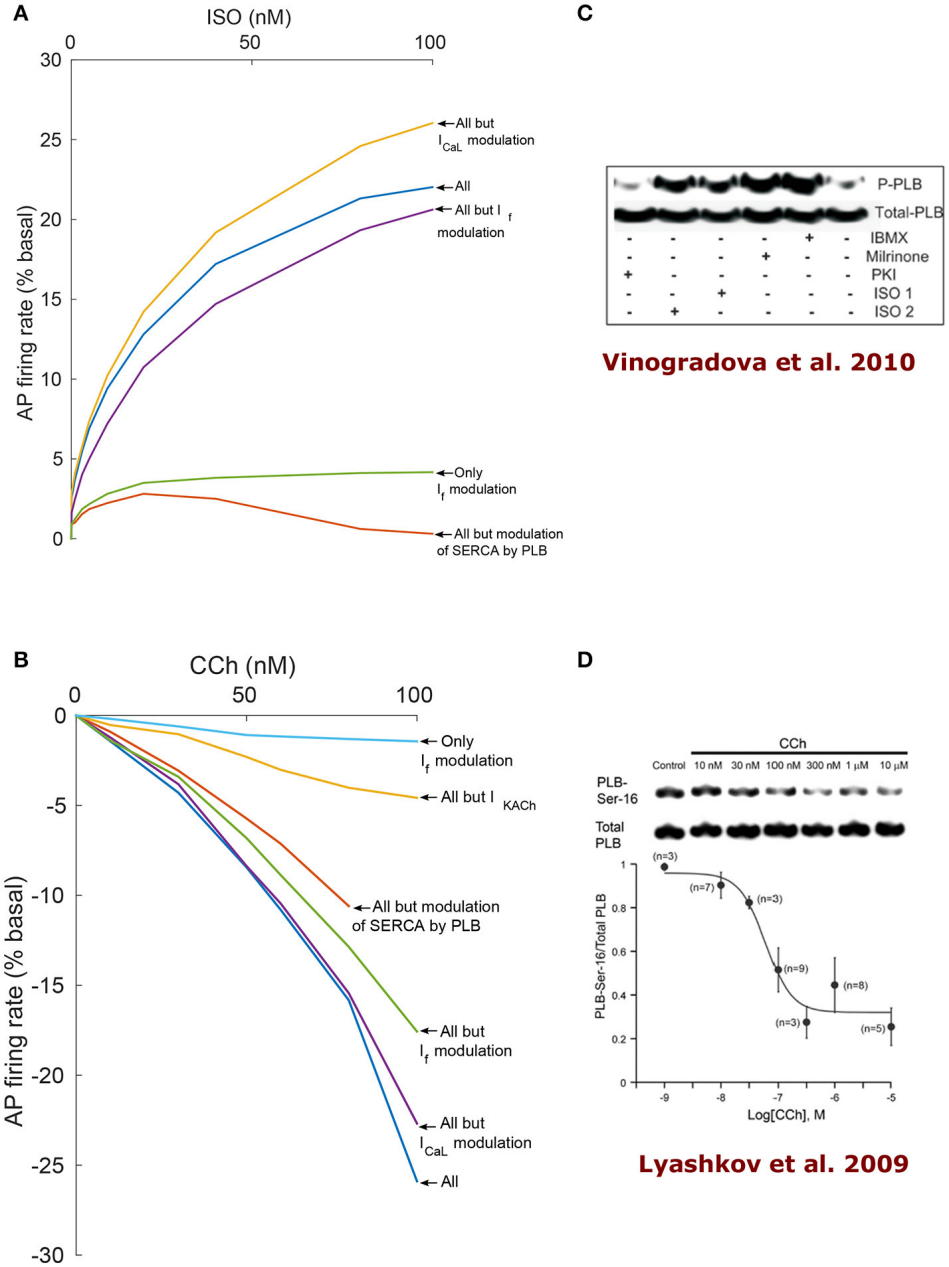
**Analysis of coupled-clock mechanisms**. Action potential firing rate (% basal) change under the effect of adrenergic receptor (β-AR) stimulation by ISO **(A)** or cholinergic receptor (ChR) stimulation by CCh **(B)**. In order to highlight the relative contribution of different system components, some mechanisms were virtually deactivated by disabling their modulation by PKA/cAMP. Of note, the RyR modulation by PKA is very minor in the current formulation of the model and thus is left intact for all the runs. SERCA: sarcoplasmic reticulum Ca^2+^ ATPase, PLB: phospholamban. **(C)** Representative western blots of PLB phosphorylated at serine^16^ site and total PLB in rabbit SANC in the basal state and following milrinone (50 μM), phosphodiesters inhibitor (IBMX, 100 μM), β-AR stimulation [0.1 μM (ISO1) or 1 μM (ISO2) isoproterenol], and PKA inhibitor (PKI, 10 μM; reproduce from Vinogradova et al., 2010). **(D)** Representative western blots of PLB phosphorylated at serine^16^ site and total PLB in rabbit SANC in the basal state and following graded concentrations of CCh (Lyashkov et al., 2009).

To find the dominant pacemaker mechanisms that mediate autonomic regulation crosstalk via cAMP- PKA signaling, we performed numerical simulations where some specific SR or membrane proteins were made PKA-phosphorylation insensitive (i.e., their degree of phosphorylation does not change under adrenergic or cholinergic stimulation and is maintained at the basal level). Figure 6 shows the results of the model simulations when varying the level of ISO and CCh treatment. PKA-dependent phosphorylation of L-type channels was predicted to play a relatively minor role in mediating the decrease in AP firing rate under low activation of ChR, although it was observed to gain relative importance as the concentration of CCh was increased. For CCh = 100 nM, the reduction in the AP firing rate was 22.7% when disabling *I*_*CaL*_ modulation by PKA (purple curve in Figure 6B) in comparison to 25.9% with all the mechanisms active (thus a difference of 3.2%). Interestingly, under β-AR stimulation, “clamping” the phosphorylation of *I*_*CaL*_ resulted in a 4.4% increase in AP firing rate with respect to when the phosphorylation modulation of *I*_*CaL*_ was activated (yellow curve in Figure 6A).

When *I*_*KACh*_ was disabled, the reduction in AP firing rate in response to activation of ChR was significantly smaller (yellow curve in Figure 6B): for CCh = 100 nM, a 4.6% decrease was measured instead of 25.9% in the case where all mechanisms were functional (thus a difference of 21.3%). When the *I*_*f*_ activation curve shift by cAMP was disabled, the AP firing rate decreased by 17.6% for CCh = 100 nM (green curve in Figure 6B) instead of 25.9% when all the mechanisms were kept active (thus a difference of 8.3%). Moreover, it increased the AP firing rate by 20.6% under ISO = 100 nM in comparison to 22% with all the mechanisms active (thus a difference of 1.4%). When only the effect of cAMP on *I*_*f*_ was taken into account (green curve in Figure 6A and light blue in Figure 6B), the AP firing rate increased by a maximum of 4.2% under stimulation by ISO and decreased by a maximum of 1.4% under stimulation by CCh. When the modulation of PLB by PKA-dependent phosphorylation was disabled, the changes in the spontaneous AP firing rate of SANCs was insensitive to β-AR stimulation (orange curve in Figure 6A). In the case of ChR stimulation and when disabling PKA-dependent phosphorylation of PLB, the model became unstable after 80 nM of CCh (orange curve in Figure 6B). Thus, the model predicts that PLB phosphorylation is a critical mechanism in the mediation of AP firing rate changes in response to either β-AR or ChR stimulation.

**Figure 7:**
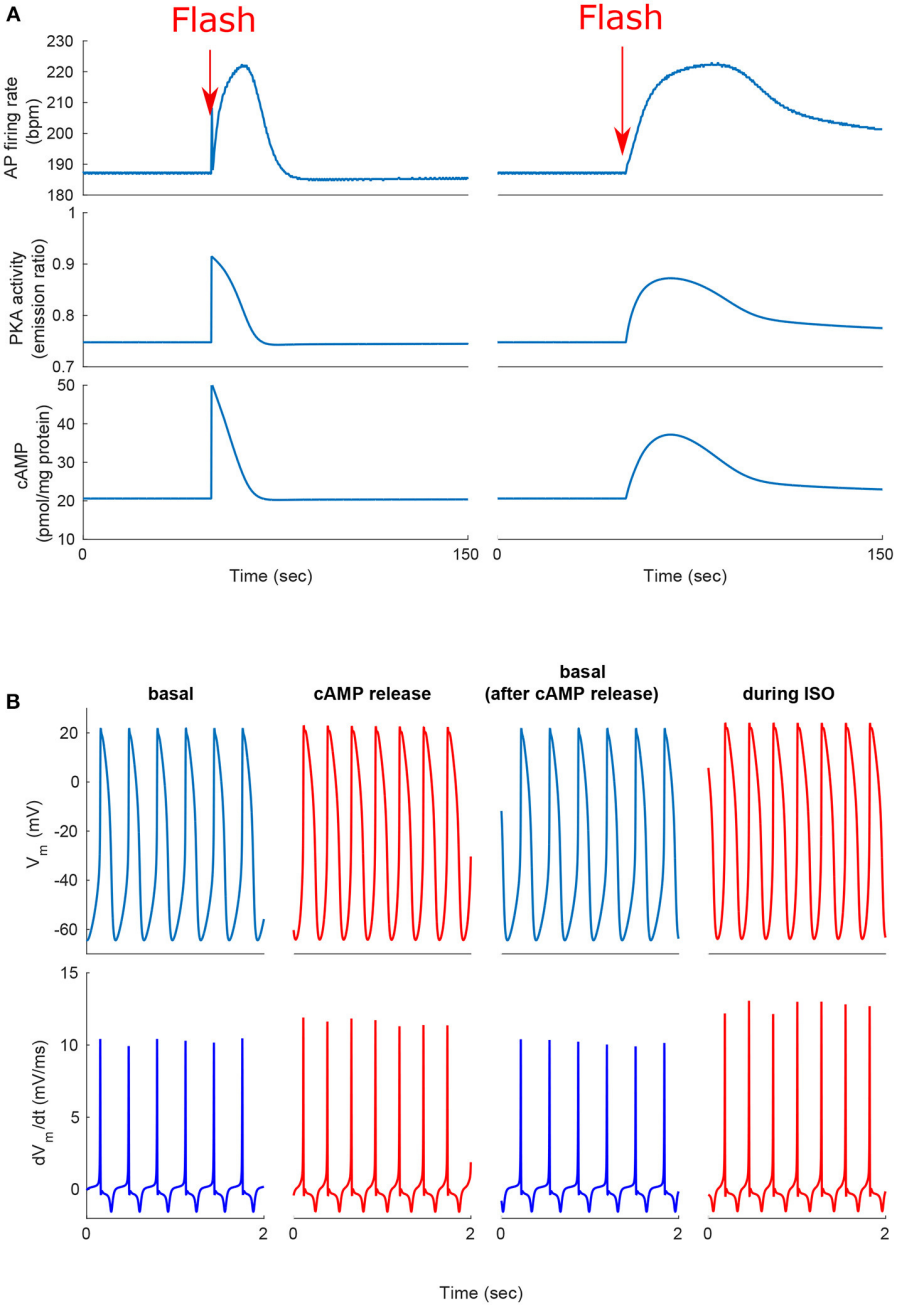
**Positive chronotropic response of SANCs to flash photolysis of caged ISO or caged cAMP**. **(A)** Change in action potential (AP) firing rate, cAMP, and PKA activity after release of the caged cAMP following the flash at *t* = 50 s. During the same cycle, the AP firing rate instantaneously increased upon flash photolysis of caged-cAMP or with flash photolysis of caged ISO. **(B)** Action potential and *dV*_*m*_/*dt* before, during, and after release of the caged cAMP or caged ISO for a 2 s segment. Notice the higher number of cycles and higher maximum *dV*_*m*_/*dt* during cAMP release or ISO treatment (in red).

**Figure 8:**
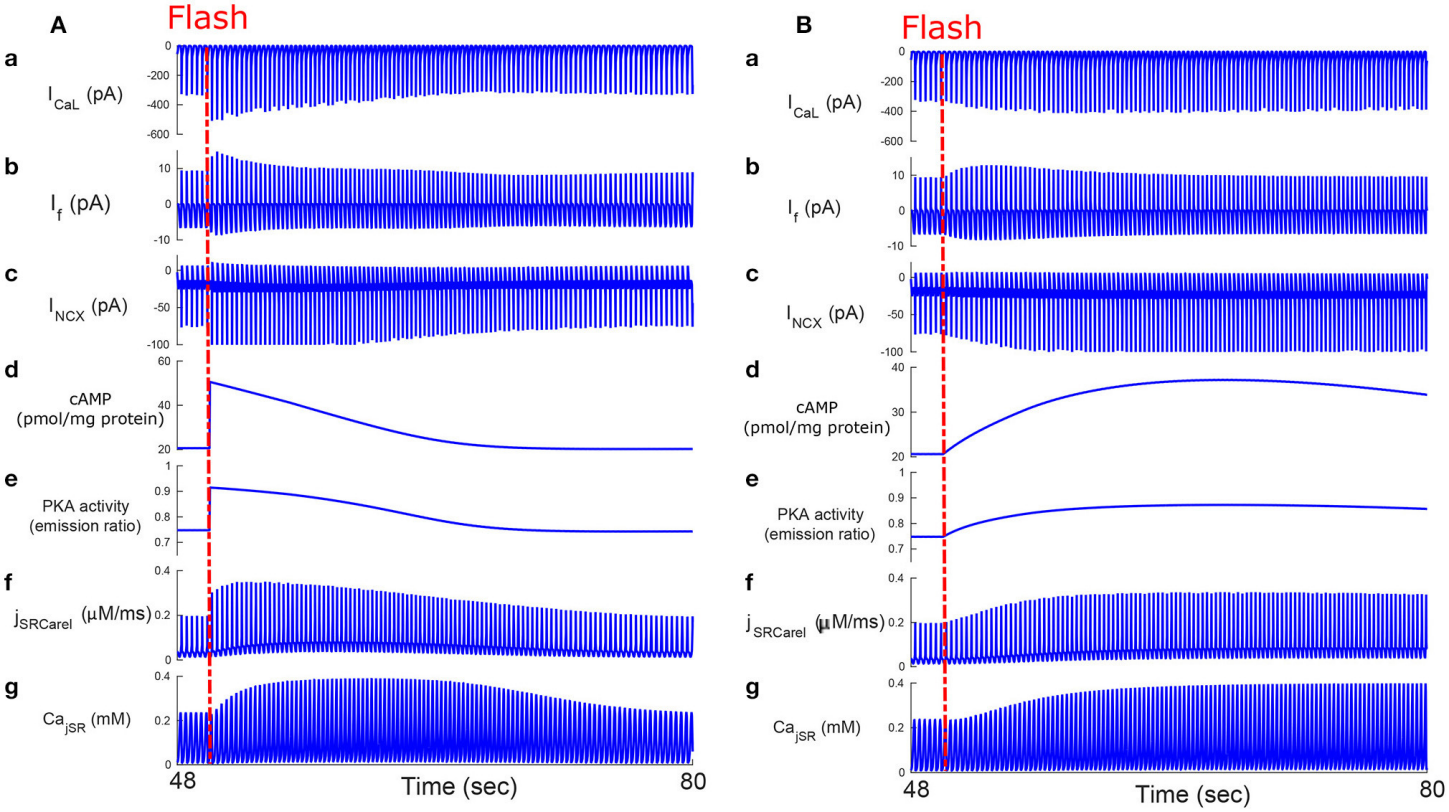
**Caged cAMP and ISO experiments**. Effect of caged cAMP **(A)** and ISO **(B)** on cellular currents: main membrane currents **(a–c)**, AC-cAMP-PKA signaling **(d,e)**, and Ca^2+^ cycling (flux and concentration) in the sarcoplasmic reticulum before and during cAMP release from the cage **(f,g)**. Notice the increase in the Ca^2+^ released from the RyR (*j*_*SRCarel*_, **f**) and of the Ca^2+^-Na^+^ exchanger (*I*_*NCX*_, **c**) after cAMP release from the cage. *I*_*CaL*_, L-type current; *I*_*f*_, funny-current; *I*_*NCX*_, the Na^+^-Ca^2+^ exchanger current; *Ca*_*jSR*_, the junctional sarcoplasmic reticulum Ca^2+^ concentration.

We next tested whether, similar to autonomic modulation, direct alteration of intracellular AC-cAMP-PKA signaling resulted in changes in the magnitude and kinetics of the AP firing rate. Specifically, we wished to compare the kinetic responses of rapid changes in either neural stimulator of AC and Ca^2+^-calmodulin activated AC. To this end, we compared the changes in the dynamics of internal pacemaker mechanisms in response to internal stimulation by release of caged cAMP (50 μM) or to autonomic receptor stimulation by release of caged-ISO (3 μM). In contrast to perfusion with ISO, the release of cAMP from caged molecules produced an immediate response. Our model simulations were compared to the only reported experimental results on the effect of caged-cAMP or ISO on the AP firing rate (Tanaka et al., 1996). Figure 7A, shows the effect before, during, and after caged cAMP release, as well as before, during, and after application of ISO. During the same cycle, the AP firing rate instantaneously increased by 19% upon flash photolysis of caged-cAMP and by up to 19% with flash photolysis of caged ISO. Figure 7B shows a snapshot of action potential membrane voltage (V_*m*_) and dV_*m*_/dt before and after flash release of caged cAMP as well as during caged-ISO release; in particular it can be observed that (dV_*m*_/dt)_*max*_ increased after flash photolysis, thus showing that early DD was initiated more quickly after cAMP release. The same observation can be made for (dV_*m*_/dt)_*max*_ during caged-ISO release. Figure 8 shows the main membrane currents (A-C), AC-cAMP-PKA signaling (D-E), and Ca^2+^ cycling (flux and concentration) in the sarcoplasmic reticulum (F-G) before and during cAMP release (Figure 8A) or ISO release (Figure 8B) from the caged molecule. In particular, there were important increases in the amount of Ca^2+^ released from RyR (*j*_*SRCarel*_, Figure 8f) and in the Ca^2+^-Na^+^ exchanger current (*I*_*NCX*_, Figure 8c) after cAMP release from the cage. Shortly after the flash photolysis of caged cAMP, the different fluxes, currents and ion concentration revert to their basal values.

**Figure 9:**
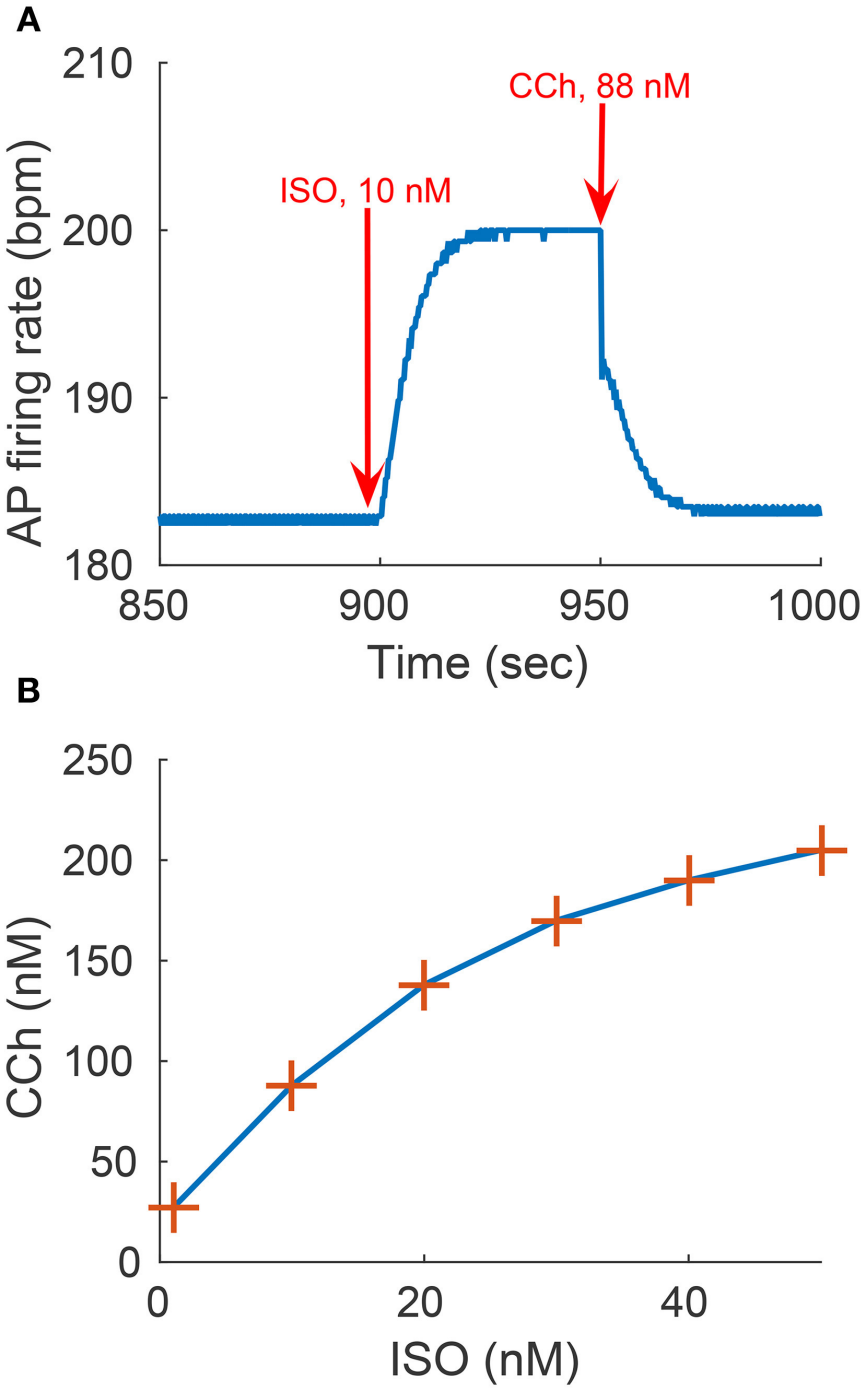
**Sympathovagal compensation**. Compensatory effects of cholinergic receptor (ChR) stimulation by CCh to adrenergic receptor (β-AR) stimulation by ISO. **(A)** Trend of the sympathovagal compensation curve, i.e., the concentrations of ISO and CCh that compensate for each other's effects on the action potential (AP) firing rate; **(B)** An example of β-AR stimulation by ISO starting at *t* = 900 s (10 nM) followed by ChR stimulation by CCh (88 nM) starting at *t* = 950 s. The AP firing rate returns to its basal rate after the addition of CCh.

**Figure 10:**
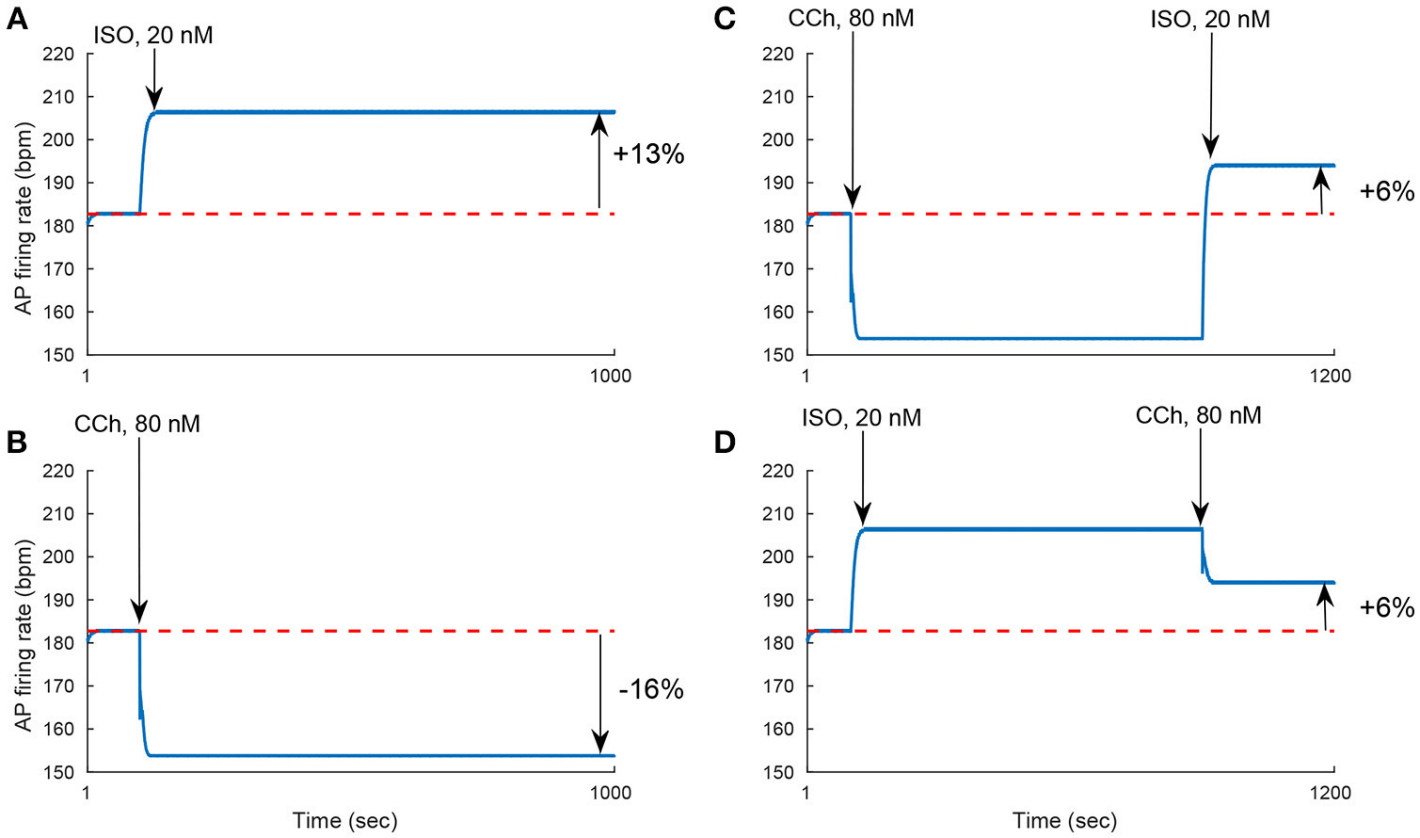
**Non additivity of adrenergic and cholinergic stimulation. (A)** Simulating the response to 20 nM ISO. The AP firing rate increases by 13%. **(B)** Simulating the response to 80 nM of CCh. The AP firing rate decreases by 16%. **(C)** Simulating the response to 80 nM of CCh followed by the addition of 20 nM of ISO. The AP firing rate increases by 6%. **(D)** Simulating the response to 20 nM of ISO followed by 80 nM of CCh. The AP firing rate increases by 6%. These set of simulations show that the response to treatment by ISO and CCh is not equal to the simple addition of the individual ISO and CCh effect on the AP firing rate (13–16% = −3% ≠ 6%).

**Figure 11:**
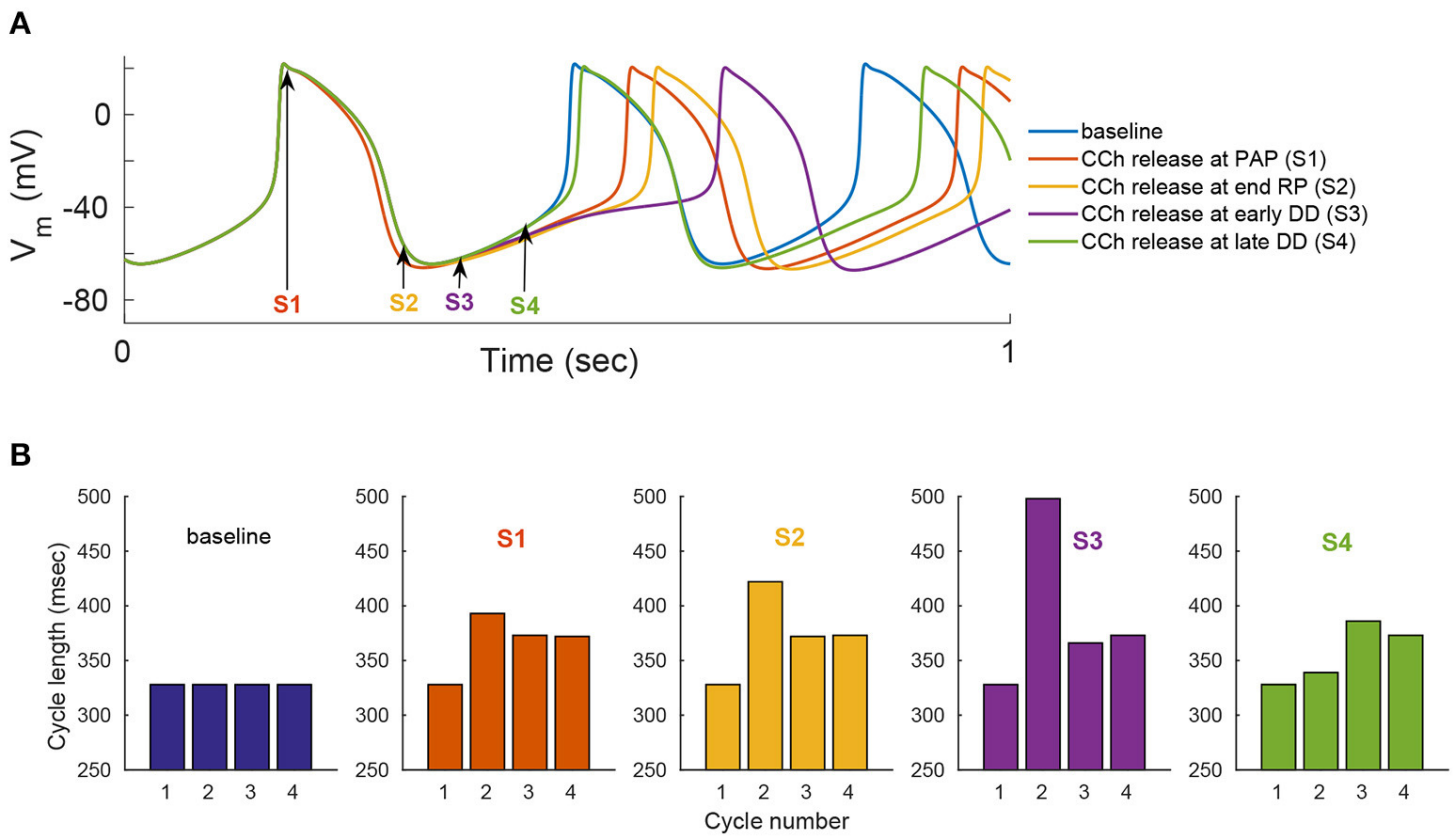
**Phase dependency of the vagal effects**. The phasic changes in pacemaker cycle length depend on the timing of the vagal stimulation during the AP cycle. To simulate this effect, flash release of 120 nM of caged CCh is simulated at different points of the action potential cycle [indicated by the arrows and markers S1–S4 in **(A)**]. Simulations when the release of caged CCh is performed at: S1, peak action potential (PAP); S2, the end of the repolarization (RP); S3, early diastolic depolarization (DD); S4, late DD. **(A)** Action potential variation as a function of caged CCh release upon flash. **(B)** Variation in cycle length for four cycles, starting one cycle before caged release of CCh. Flash lasts for 50 ms and, *C*_*b*_ = 120 nM, *k*_*CCh, off*_ = 0.11 0.3 ms^−1^, *k*_*CCh, on*_ = 10*e*−7 mM^−1^ ms^−1^.

We evaluated the model's ability to reproduce some known sympathovagal effects. The compensatory effects of ChR stimulation by CCh to β-AR stimulation by ISO are shown in Figure 9. The model predicted that 88 nM of CCh would compensate for 10 nM of ISO (thus, a ratio 10:88 for rabbit SANCs). Furthermore, the model predicted a change in this ratio in response to an increase in ISO (Figure 9B). That is, relatively less CCh is needed to compensate for increased ISO treatment. Next we evaluated whether the effect of combined CCh and ISO is additive (Figure 10). Application of 20 nM of ISO increased the AP firing rate by 13% compared to the baseline (Figure 10A), while application of 80 nM CCh reduced the AP firing rate by 16% compared to the baseline (Figure 10B). However, when CCh was applied before (Figure 10C) or after ISO (Figure 10D), the net effect of both drugs was a 6% increase in the AP firing rate (i.e., not additive). Finally, we tested how and whether rapid application of CCh has a phasic effect on the AP firing rate (Figure 11). Application of short CCh pulse (see Section Methods for further details) has a phasic effect on the AP firing rate. The phasic changes in pacemaker cycle length depend on the timing of the vagal stimulation during the AP cycle. The greatest effect on AP cycle (appeared on the following beat) was observed when CCh was applied during early DD (Figure 11, see S3).

## Discussion

Our first and most important major conclusion is that AC-cAMP-PKA signaling crosstalk autonomic activity to SANC function (Figures 1, 4). Our experimental data showed a tight correlation between the spontaneous AP firing rate and the PKA activity level in response to autonomic receptor stimulation (Figure 3). Using numerical modeling, we predict, for the first time, that in response to autonomic regulation (sympathetic and/or parasympathetic) of SANCs, changes in the cAMP/PKA activity level will lead to changes in SANCs function. This is because the change in the PKA activity level will modulate the activation of critical membrane and SR proteins and changes in the cAMP level will modify the *I*_*f*_ activation curve. These cAMP/PKA induced changes will themselves modify the function of the coupled clock. Furthermore, our numerical simulations predict that eliminating the effect of cAMP and PKA or PKA only will reduce or neutralize the changes in the spontaneous AP firing rate in response to autonomic receptor stimulation (Figure 6). Of note, our model simulation predictions are in good agreement with the experimental results obtained here (Figure 3). Moreover, when running the model for a large range of β-AR (0–100 nM) or ChR (0–100 nM) concentrations, the AP firing rate-cAMP relationship is coherent with experimental data obtained using drugs that perturbed internal cell mechanisms (Figure 5). Because the current model includes descriptions of both clock mechanisms, post-translation modification signaling cascades, and autonomic-nervous receptors, it should be useful to predict pathological alterations in the function of any of these components. Of note, a recent paper using mice as an experimental model found that PKA signaling played a minor role under basal conditions (Wu et al., 2016). The difference between the current and Wu et al. results might be related to the mammal each group is working with. Alternatively, it might be related to other non-specific gene changes in these mutant mice (e.g., L-type) that contribute to AP firing.

Our second major conclusion is that PLB phosphorylation is the dominant mechanism mediating between autonomic receptor stimulation and SANCs function. Disabling the modulation of PLB by PKA- dependent phosphorylation leads to insensitivity to β-AR stimulation (Figure 6A). In the case of ChR stimulation, disabling PKA-dependent phosphorylation of PLB results in a higher AP firing rate and causes the model to become unstable after 80 nM of CCh (Figure 6B). This result may be a model artifact or a physiological phenomenon that must be tested experimentally (e.g., by reducing PKA-dependent phosphorylation by cyclopiazonic acid and testing the effect of CCh). The predictions of the model regarding the critical role of the SERCA pump are in accordance with the results of Itzhaki et al. (2011), who demonstrated the importance of Ca^2+^ reuptake into the SR via the SR Ca^2+^ ATPase (SERCA) pump in human-induced pluripotent stem cell derived cardiomyocytes. The authors used thapsigargin, a SERCA inhibitor that leads to the elimination of Ca^2+^ local calcium release. Moreover, in SANC, the suppression of the phosphorylation level of PLB was associated with the suppression of the spontaneous beating rate (Vinogradova et al., 2006). This experiment (Vinogradova et al., 2006) shows that the phosphorylation level of PLB (see Figures 6C,D) (and thus the activation level of the SERCA pump) is associated with cholinergic stimulation. *I*_*KACh*_ was also found to be critical under ChR stimulation, which is coherent with the experimental findings of Van Borren et al. (2010), who showed that *I*_*KACh*_ plays an important role in the acetylcholine-mediated AP firing rate decrease in SANCs. *I*_*f*_ -cAMP dependent modulation of the AP firing rate was found to be higher when the dose of ISO or CCh was increased. This is consistent with the fact that higher doses of ISO (or CCh) cause the cAMP level to increase (decrease) nonlinearly, resulting in a higher cAMP-dependent shift in the *I*_*f*_ activation curve (see Figure S5) and thus a greater change in the *I*_*f*_ current. Under both β-AR or ChR stimulation, the effect of cAMP activation of *I*_*f*_ alone on the AP firing rate was found to be relatively minor (Figure 6), in agreement with the fact that β-AR or ChR stimulation do not produce significant changes in *I*_*f*_ amplitude relative to other currents (*I*_*NCX*_ and *I*_*CaL*_; see Figure 4). This finding suggests that the cAMP-induced *I*_*f*_ activation curve shifts are not the main mediator of the chronotropic effect simulated under autonomic regulation. Indeed, relative to the role of PKA in modulating membrane and SR protein targets, direct cAMP modulation of membrane targets plays a less dominant role in regulating the AP firing rate. Interestingly, under β-AR stimulation, PKA-dependent phosphorylation of L-type channels was shown to actually decrease the AP firing rate (blue curve, Figure 6A) with respect to when this mechanism was deactivated (yellow curve, Figure 6A). Our model simulation predicts that this effect is due to inactivation of the Ca^2+^ mechanism in this channel: under β-AR stimulation, higher LCR from the RyR channels together with the higher phosphorylation-dependent activation of the L-type channels will increase the Ca^2+^ in the submembrane space, which will activate the Ca^2+^ inactivation gate of *I*_*CaL*_ (*f*_*CaL*_; see S.3.3.1 in the Supplementary Material), which will eventually reduce its effective current. Figure S6 shows how the AP, the *I*_*CaL*_ Ca^2+^ dependent inactivation gating variable, and the subsarcolemmal Ca^2+^ concentration vary in accordance with whether the phosphorylation effects on *I*_*CaL*_ are taken into account. It can be observed in Figure S6C that the lower activation of *I*_*CaL*_ when the phosphorylation dependency is not present leads to a reduction in Ca^2+^ cycling inside the cell. This in turn leads to a lower subsarcolemmal Ca^2+^ concentration and thus less inactivation of the Ca^2+^ dependent gate (Figure S6B). Conversely, if the phosphorylation effects on *I*_*CaL*_ are taken into account, then more Ca^2+^ cycles (Figure S6C) leading to increased inactivation of the *I*_*CaL*_ Ca^2+^ dependent gate (Figure S6B), which results in lowering the AP firing rate (Figure S6A). Of note, the model predictions in that respect are in contradiction with the simulation results in Severi et al. (2012), who showed that inactivation of the phosphorylation effect on *I*_*CaL*_ resulted in a lower AP firing rate upon adrenergic stimulation. This is likely due to the different emphasis on the role of Ca^2+^ cycling vs. the contribution of *I*_*f*_ between the two model types. Thus, the model predicts that above a certain increase in the PKA activity level, the phosphorylation of L-type channels acts as a brake to compensate for the Ca^2+^ increase in the submembrane space. Because patch clamp experiments are performed under constant Ca^2+^, the Ca^2+^ inactivation effect cannot be measured directly and thus can only be quantified by numerical modeling. The role of L-type channels in mediating changes in spontaneous AP firing rate in response to β-AR stimulation is thus not in accordance with Tanaka et al.'s (1996) conclusion (see Section Discussion in the following paragraph).

Our third major conclusion is that direct disturbance of either the internal pacemaker clock mechanisms or the autonomic receptors leads to changes in the spontaneous AP firing rate, mainly through AC-cAMP-PKA signaling. We compared the changes in the dynamics of internal pacemaker mechanisms induced by internal stimulation by release of caged cAMP (50 μM) to the changes induced by release of caged ISO (Figure 7). We also compared the relative kinetic and chronotropic effects of caged cAMP release as opposed to caged ISO. We observed that the increase in the AP firing rate was faster for the caged cAMP simulation than it was for the caged ISO, similar to the experimental results of Tanaka et al. (1996). Given that β-AR stimulation is external and initiates biochemical pathway cascades that involve AC-cAMP-PKA signaling, we interpret this result to mean that changes in the AP firing rate are more rapidly induced by internal than by external stimulation, and thus, that the kinetics for the conversion of β-AR stimulation into cAMP signaling are slower than the subsequent kinetics of cAMP-dependent phosphorylation of membrane and SR proteins. However, despite the different kinetics for β-AR stimulation and caged cAMP release, the effects on the membrane ion channels and SR receptors were similar (see Figure 8). This further highlights that β-AR stimulation is mediated by cAMP signaling. Flash photolysis of caged cAMP resulted in an increase of the AP firing rate and *I*_*CaL*_, similar to the findings of Tanaka et al. (1996). However, we also report changes in other coupled-clock mechanisms mediated by changes in AC-cAMP-PKA signaling. We interpret this to mean that L-type channel phosphorylation is not the major mechanism behind the changes in the spontaneous AP firing rate in response to external or internal stimulation (Figure 8). Of note, in the caged cAMP simulation, the AP firing rate increased by 19%, whereas in Tanaka et al. the increase was reported to be, on average, from 97.4 to 153 bpm (i.e., an increase of 57%). This difference can be explained by the particularly low SANC AP firing rate in the Tanaka experiments. Indeed, 97.4 bpm does not fall in the physiological range of spontaneous AP firing rate for fresh rabbit SANCs (27). In summary, the caged cAMP simulation highlights the causal link between cAMP increase and the resulting change in the AP firing rate. The experimental results from Figure 3 shows the association between PKA activity and the AP firing rate and Figure 5 shows how the changes in AC-cAMP-PKA signaling under β-AR or ChR stimulation accurately predicted the changes in AP firing rate with respect to experimental data. In short, the experiments and simulations provide evidence that pacemaker cell function is regulated by the ANS (and thus the resulting AP firing rate) via AC-cAMP-PKA signaling under adrenergic stimulation. The model also showed that the *I*_*KACh*_ pathway was critical to the changes in the AP firing rate under cholinergic stimulation (Figure 6). These results are consistent with recent findings on GIRK4^−/−^ mice (Mesirca et al., 2013). Thus, *I*_*KACh*_ together with AC-cAMP-PKA mediated changes could explain the cholinergic effects on the AP firing rate.

Our fourth major conclusion is that the sympathovagal compensation ratio (the balance between sympathetic and parasympathetic stimulation) varies under different regimes of AP firing rate (Figure 9). The model predicts that this ratio will change as ISO is increased (Figure 9B): relatively less CCh will be needed to compensate for increased ISO treatment. The model also predicts that the kinetics of the ISO response to β-AR stimulation are much slower than the CCh response to ChR stimulation (Figure 9). This can be explained by the fact that the ChR response is affected not only by AC-cAMP-PKA signaling but also by the faster kinetics of the activation of the muscarinic current, *I*_*KACh*_. To the best of our knowledge, there are no experimental results for fresh rabbit SANCs to which these results can be compared. Of note, a higher value of CCh was needed to compensate for ISO stimulation in the experiments presented in Figure 3: 1000 nM of CCh was insufficient to compensate for 1 nM ISO. However, PKA activity was measured in cultured SANCs (see Section Methods and Supplementary Material), whereas the numerical model is built for fresh rabbit SANCs. Thus, the compensatory effects of CCh are not expected to be of the same order of magnitude. In addition, the set of simulations presented in Figure 10 shows that the response to simultaneous ISO and CCh cannot be explained merely by additive effects of ISO and CCh. This is in accordance with experimental findings (Levy, 1971). The non-additive effect in the current model is related to the non-linear relationship between cAMP vs. AP firing and not to the direct crosstalk between β-AR and ChR. Future experiments will have to define which of the two mechanisms is the dominant one and whether it exists in SANCs. Note that neither the MDP or the AP amplitude changed significantly in response to ISO or in response to a combination of ISO and CCh (Figure S7). Finally, the simulation in Figure 11 shows that the model is able to reproduce some aspects of the well-known phase dependency of the vagal effects. Similar to the results of Jalife and Moe (1979), the first beat was delayed maximally when the CCh release was applied later (S2–S3) (thus delaying the ChR stimulation effect) than when it was applied at the peak action potential (S1). In our simulation, the delayed first beat was greatest when the CCh release started during the early DD (S3).

Figure 5 shows the relationship between AP firing rate and cAMP under varying the degrees of autonomic stimulation as predicted by the model. The black crosses denote experimental data published in previous work (Yaniv et al., 2013d), where the concentration of cAMP and the associated AP firing rate were measured in response to a battery of pharmacological drugs. The predicted trend was very similar to the trend exhibited in the experimental results (see Figure 5). Furthermore, the predicted AP firing rate in response to CCh (100 nM) and ISO (100 nM) fell in the range of our new experimental results (see Figure 3). For high concentrations of ISO, the increase in AP firing rate was in the range of previously reported experimental studies for cultured and fresh cells: a 20–35% increase (Rigg et al., 2000; Vinogradova et al., 2002; Yaniv et al., 2013d, 2015a).

To verify that the current model produced a coherent AP waveform and coherent current characteristics, we compared its parameters to other existing model parameters. Table 1 summarizes the traditional AP characteristics for state-of-the-art numerical SANCs models and our new model. The new model statistics are within the range of previously published experimental data and similar to those of previously published models (Noble and Noble, 1984; Demir et al., 1994; Zhang et al., 2000; Kurata et al., 2002; Maltsev and Lakatta, 2009; Severi et al., 2012; Yaniv et al., 2015a). Table 2 offers a comparison of some key current characteristics from existing models and our new model. Our new model features a late diastolic *I*_*NCX*_ of the order of magnitude of the ML model (Maltsev and Lakatta, 2009). However, the maximum diastolic *I*_*f*_ was higher in our model than in the coupled-clock models of ML and YL (Maltsev and Lakatta, 2009; Yaniv et al., 2015a). Finally, the simulations also showed the presence of some low beat-to-beat amplitude oscillations in the PKA and cAMP level. This is due to the calmodulin-activated AC, which varies with the calmodulin buffering level and thus with Ca^2+^ cycling. The presence, amplitude, and role of PKA oscillation at steady state has not been experimentally studied in pacemaker cells. In the simulation in Figure 4, small changes in the maximal diastolic potential can be observed upon application of ISO or ACh. However, these changes are difficult to quantify experimentally. In Bucchi et al. (2007) the authors showed that no significant changes in MDP could be observed upon application of low and high concentration of ISO. When applying a high concentration of ACh, a small reduction of the MDP could be observed (Bucchi et al., 2007), in accordance with the predictions of the model (see Figure 4).

**Table 1:** **Traditional action potential characteristics for state-of-the-art numerical models of rabbit SANCs and the present model in its basal state**.

**Statistic/model**	**Noble and Noble, 1984**	**Zhang et al., 2000**	**Demir et al., 1994**	**Kurata et al., 2002**	**Maltsev and Lakatta, 2009**	**Severi et al., 2012**	**Yaniv et al., 2015a**	**Current model**	**Experimental range**
MDP, mV	−61	−58	−61	−59	−63	−58	−63	−64	−56 ± 6 (−66 ÷ −52)
APA, mV	84	79	96	75	76	80	78	86	87 ± 6 (78 ÷ 98)
CL, ms	263	327	263	308	333	352	324	328	325 ± 42 (247 ÷ 389)
dv/dt_*max*_, V/s	4.7	2.7	9.6	6.4	4.8	7.1	6.1	10.2	11.3 ± 6.5 (4.8 ÷ 27)

*MDP, maximum diastolic potential; APA, action potential amplitude; CL, cycle length; dv/dt_max_, maximum rate of rise of membrane potential during AP upstroke. Experimental ranges are reported as in Severi et al. (2012)*.

**Table 2:** **Characteristic amplitude of ion currents that contribute to mid to late diastolic depolarization (DD)**.

**Statistic/model**	**Noble and Noble, 1984**	**Zhang et al., 2000**	**Demir et al., 1994**	**Kurata et al., 2002**	**Maltsev and Lakatta, 2009**	**Severi et al., 2012**	**Yaniv et al., 2015a**	**Current model**
Maximum diastolic *I_f_*, *pA/pF*	0.061	0.1	0.073	0.109	0.068	0.2	0.123	0.196
Maximum *I*_*CaT*_, *pA/pF*	—	0.353	0.188	0.227	0.09	0.19	0.086	0.120
Maximum peak *I*_*CaL*_, *pA/pF*	4.95	3.46	11	6.87	5.35	6.0	6.687	10.44
Late diastolic *I*_*NCX*_, *pA/pF*	0.06	0.03	0.15	0.328	0.458	0.21	0.550	0.521

*Table adapted from Maltsev and Lakatta (2009) by adding the data for the most recent models from Severi et al. (2012), Yaniv et al. (2015a), and our new model. Late diastolic Na^+^—Ca^2+^ exchanger current I_NCX_ was measured at 85% of cycle length from the upstroke at 0 mV of the prior action potential (Maltsev and Lakatta, 2009). The statistics for the Severi et al. model were measured on Figure 6 of their paper (Severi et al., 2012)*.

Despite the numerous studies on SANC modeling, to the best of our knowledge only three were found to include the modeling of autonomic modulation (Demir et al., 1999; Himeno et al., 2008; Maltsev and Lakatta, 2010). (a) Maltsev and Lakatta (2010) modeled the effects of autonomic regulation in a phenomenological manner, i.e., without explicitly modeling the AC-cAMP-PKA signaling sequence and the effect of the cAMP and PKA levels on the phosphorylation of the SR and membrane proteins. (b) Himeno et al. (2008) introduced a guinea pig SANC model that included response to adrenergic stimulations and explicitly modeled the AC-cAMP-PKA signaling sequence and its activation of key SR and membrane proteins. However, the model did not include cholinergic stimulation. Moreover, the enhanced Ca^2+^ release from the SR failed to induce an important chronotropic effect due to the model's exclusion of the subsarcolemmal space, which resulted in a very low increase in intracellular Ca^2+^ concentration during local calcium release and thus a lower Ca^2+^ activation of *I*_*NCX*_. In addition, the Himeno et al. (2008) model identified that cAMP-PKA modulation of *I*_*st*_ was a main contributor to the chronotropic effects. However, *I*_*st*_ is thought to represent a surrogate of other currents (*I*_*CaL*_ and *I*_*NCX*_) (Maltsev et al., 2014) as it exhibits many properties of these currents; it is thus unlikely that *I*_*st*_ modulation is a dominant mechanism. Finally, the modeling of Ca^2+^ activated AC as used in our model (see Figure 2, k_1_) was missing. (c) In Demir et al. (1999) the following ion channels were modulated by autonomic stimulation: *I*_*KAch*_, *I*_*Na*_, *I*_*b, Na*_, *I*_*CaL*_, *I*_*K*__,_ *I*_*NaK*,_ and *I*_*f*_. The model explicitly includes the cAMP balance using a differential equation and modeled the autonomic effects on the ion channels as a function of the cAMP level for the following currents: *I*_*f*_, *I*_*CaL*,_ and *I*_*K*_. However, it is known that the *I*_*CaL*_, *I*_*K*_ currents are modulated by PKA dependent phosphorylation and not directly by the cAMP level. In addition, as pointed in Maltsev and Lakatta (2010), this model (Demir et al., 1999) is based on an anterior model for which the Ca^2+^ activation was triggered by the membrane depolarization. This implicitly assumes the preponderance of the M-Clock over the Ca^2+^ clock in SANC function, in contradiction to some recent studies (Yaniv et al., 2013b,e). Thus, this model was limiting in that the modulation by PKA of key SR proteins (SERCA and RyR) could not be studied. In addition, a number of current formulations were adapted from ventricular cell models. In conclusion, the previous SANC models that included autonomic modulation all had limitations that we addressed in this work by: (a) modeling both cholinergic and adrenergic effects; (b) explicit modeling of AC-cAMP-PKA signaling; (c) using the most recent modeling of the Ca^2+^ activated *I*_*NCX*_ current to take into account the coupling between the M and Ca–clocks; (d) Ca^2+^ activated AC.

In the rabbit SANC model from Severi et al. (2012), *I*_*f*_ was the main contributing current to the early diastolic depolarization. Indeed, the main assumption within the Severi et al. model is that *I*_*f*_ is the major inward ionic current until late DD and thus the main regulator of the AP firing rate. This is different from the family of models we built upon, which hypothesize that Ca^2+^ handling is the main regulator of the AP firing rate. Severi et al. also simulated the effect of autonomic modulation using a phenomenological approach, resulted in a 19.6% AP firing rate decrease under cholinergic stimulation (10 nM of ACh) and to a 28.2% AP firing rate increase under 1 μ*M* of ISO. These predictions are in the range of our model prediction under a high concentration of CCh and ISO.

In a recent work (Behar and Yaniv, 2016) we highlighted how a modification of AC-cAMP-PKA signaling could produce effects similar to those of ivabradine (which is used to block funny channels), and might thus be used to eliminate AP firing rate tachycardia caused by a mutation in *I*_*f*_. Modulation of intracellular cAMP/PKA affects SANC function by targeting the coupled-clock mechanisms rather than targeting a specific molecule. Note, that although ivabradine is a specific drug it has unspecific effects on both clocks (Yaniv et al., 2013b). A better understanding of how the level of cAMP/PKA is regulated could pave the way for an alternative to ivrabradine for tachycardia treatment and open new pathways to bradycardia treatment. In other words, control of the cAMP/PKA activity level by either or the combination of internal mechanism blockers (e.g., IBMX) or/and autonomic-nervous receptors (e.g., β-blockers) could be an appropriate therapeutic goal for treating heart rhythm disturbances.

## Limitations

Our new model has several limitations. First, although its 34 coupled differential equations (not including the differential equations for the caged cAMP/ISO experiments) integrate many concepts and experimental findings, the experimental results used to build the model are taken from the literature. These experimental results are not always obtained under the same conditions (e.g., fresh vs. cultured cells) or on the same mammal. In addition, due to technical limitations, our new experiments for quantifying PKA activity and AP firing rate were performed on different groups of SANCs. Measuring a number of physiological quantities (e.g., PKA, cAMP, and AP firing rate) simultaneously on a set of SANCs would allow us to improve the predictive accuracy of the model. Second, although the modulation of RyR by PKA-dependent phosphorylation was taken from Yaniv et al. (2015a) (see Supplementary Material), no experimental results quantifying RyR phosphorylation by PKA exist for rabbit SANCs. Thus, it might be necessary to refine the model when new experimental data on RyR phosphorylation by PKA are obtained. Third, although the role of Ca^2+^ -activated potassium channels (Haron-Khun et al., 2016) and early L-type opening channels (Mangoni et al., 2003) have been documented in mice, their roles in rabbits are not clear.

Other limitations of the model include the lack of explicit modeling of the CaMKII-dependent phosphorylation of membrane and SR proteins due to the lack of experimental data on rabbit SANCs and the limited working range (100 nM) for CCh treatment. Therefore, it is possible that some mechanisms activated by ChR receptor stimulation are missing from our model. Note however, that CaMKII and PKA signaling crosstalk (Yaniv et al., 2013c). Therefore, the results of the effect of PKA on SANC function imply on the role of CaMKII signaling. Future experiments on CaMKII activity by using similar approach used here to measure PKA signaling is needed. The model also needs to be improved to be able to reproduce the effect of autonomic modulation for a higher range of CCh. In addition, the relative contribution of the funny current to the AP firing rate with respect to other early DD currents is still open to discussion (Verkerk and Wilders, 2013). In our model the AP firing rate was reduced by 13% when disabling *I*_*f*_ and running the model under basal conditions. A partial (70%) block of the *I*_*f*_ channels resulted in a 7.4% decrease in the AP firing rate, which is in the experimental range (5–30%; Denyer and Brown, 1990; Vinogradova et al., 2002; Difrancesco, 2005; Bucchi et al., 2007). This simulation was performed as a complement to the total *I*_*f*_ block simulations presented in Figure 5 because it has been shown experimentally that using funny channel blockers results in only a partial block of these channels. Note that, despite these limitations, the model managed to fit experimental data fairly well for a large range of β-AR or ChR stimulation levels (Figures 3, 5, Tables 1, 2). Finally, the model is limited by the lack of experimental values for *k*_*cAMP, on*_ and *k*_*ISO, on*_, that is, the lack of experimentally evaluated values for the rates of cAMP and ISO caging. These values were taken to be small, so that the buffering kinetics would be slow, as described in the literature. Although, it was not possible to use experimental estimates for these two constants, the model managed to reproduce the expected experimental behavior (Figure 7).

## Conclusions

The present work provides the first experimental and theoretical evidence that the ANS is the dominant regulator of pacemaker cell function (and thus the resulting AP firing rate) via AC-cAMP-PKA signaling and the activation of the muscarinic current under cholinergic stimulation. Because AC-cAMP-PKA signaling acts as a messenger that modulates internal pacemaker mechanisms, it allows pacemaker flexibility and robustness. The numerical model predicts that the activation of the SERCA pump via PLB phosphorylation is the main player in mediating the change in AP firing rate within the autonomic modulation regulatory process of SANCs. We also identified *I*_*KACh*_ as being a major contributor to pacemaker flexibility under ChR stimulation. Experimentally, we showed that PKA activity was associated with pacemaker function in the entire physiological range of SANC AP firing rates, thus providing further evidence to validate our hypothesis. The novel model predictions motivate further experimental research for better understanding the role of AC-cAMP-PKA signaling and evaluating the importance of PLB in regulating the AP firing rate in response to autonomic receptor stimulation. It also motivates new experimental studies to assess RyR function under β-AR or ChR stimulation and the development of new experimental methods for recording a number of physiological quantities (e.g., PKA, cAMP, and AP firing rate) simultaneously. A better understanding of the role of PKA-cAMP signaling could pave the way for novel treatment solutions for heart rhythm disturbances.

## Author contributions

YY and JB conception and design of research. JB performed simulations; YY and AG performed SANC experiments; JB analyzed both simulated and experimental data; JB and YY interpreted results of experiments; JB drafted manuscript; JB prepared figures; JB, YY, and JZ edited and revised manuscript; JB, AG, JZ, and YY approved final version of manuscript.

## Funding

The work was supported by the NSFC-ISF Joint Research Program, No. 398/14 (YY), the Israel Ministry of Science Technology and Space (YY), the Ministry of Aliyah and Immigrant Absorption (JB), and an Aly-Kaufman Postdoctoral Fellowship (JB). The funders had no role in study design, data collection and analysis, decision to publish, or preparation of the manuscript.

### Conflict of interest statement

The authors declare that the research was conducted in the absence of any commercial or financial relationships that could be construed as a potential conflict of interest. The handling Editor declared a shared affiliation, though no other collaboration, with one of the authors AG and states that the process nevertheless met the standards of a fair and objective review.
