# Supplementary material for: The Autonomic Nervous System Regulates the Heart Rate through cAMP-PKA Dependent and Independent Coupled-Clock Pacemaker Cell Mechanisms
Source: Front Physiol. 2016 Sep 27;7:419. doi: 10.3389/fphys.2016.00419 (PMC5037226; doi:10.3389/fphys.2016.00419)
Supplement: Supplementary file 1 [file DataSheet1.docx]

# Supplement

## S.1 Coupled differential equation variables

|  | **Symbol** | **Initial value** | **Units** | **Description** |
| --- | --- | --- | --- | --- |
| *1* | ${{[Ca}^{2+}]}_{i}$ | 0.0001 | mM | [Ca^2+^] in myoplasm |
| *2* | ${{[Ca}^{2+}]}_{sub}$ | 0.000223 | mM | [Ca^2+^] in submembrane space |
| *3* | ${{[Ca}^{2+}]}_{jsr}$ | 0.029 | mM | [Ca^2+^] in the junctional SR (JSR) |
| *4* | ${{[Ca}^{2+}]}_{nsr}$ | 1.35 | mM | [Ca^2+^] in the network SR (NSR) |
| *5* | ${{[Ca}^{2+}]}_{m}$ | 0.00005 | mM | [Ca^2+^] in mitochondria |
| *6* | $f_{CMi}$ | 0.042 | [] | Fractional occupancy of calmodulin by Ca^2+^ in myoplasm |
| *7* | $f_{CMs}$ | 0.089 | [] | Fractional occupancy of calmodulin by Ca^2+^ in submembrane space. |
| *8* | $f_{CQ}$ | 0.032 | [] | Fractional occupancy of calsequestrin by Ca^2+^ in junctional SR |
| *9* | *R* | 0.749 | [] | RyR reactivated (closed) state |
| *10* | *O* | 3.4·10-6 | [] | RyR open state |
| *11* | *I* | 1.1·10-6 | [] | RyR inactivated state |
| *12* | *RI* | 0.25 | [] | RyR RI state |
| *13* | $V_{m}$ | -65 | mV | Membrane potential |
| *14* | $d_{L}$ | 0 | [] | *I_CaL_* voltage-dependent activation gating variable |
| *15* | $f_{L}$ | 1 | [] | *I_CaL_* voltage-dependent inactivation gating variable |
| *16* | $f_{Ca}$ | 1 | [] | *I_CaL_* Ca^2+^ dependent inactivation gating variable |
| *17* | $p_{aF}$ | 0 | [] | *I_Kr_* voltage-dependent fast activation gating variable |
| *18* | $p_{aS}$ | 0 | [] | *I_Kr_* voltage-dependent slow activation gating variable |
| *19* | $p_{ai}$ | 1 | [] | *I_Kr_* voltage-dependent inactivation gating variable |
| 20 | *N* | 0 | [] | *I_Ks_* voltage-dependent activation gating variable |
| *21* | *y* | 1 | [] | *I*_f_ voltage-dependent activation gating variable |
| *22* | $d_{T}$ | 0 | [] | *I_CaT_* voltage-dependent activation voltage-dependent |
| *23* | $f_{T}$ | 1 | [] | *I_CaT_* voltage-dependent inactivation voltage-dependent |
| *24* | *q* | 1 | [] | *I_to_* voltage-dependent inactivation voltage-dependent |
| *25* | *r* | 0 | [] | *I_to_* and *I*_sus_ voltage-dependent activation voltage-dependent |
| 26 | $q_{a}$ | 0 | [] | *I_st_* voltage-dependent activation voltage-dependent |
| 27 | $q_{i}$ | 1 | [] | *I_st_* voltage-dependent inactivation voltage-dependent |
| 28 | *W* |  | [] | *I_KACh_* ACh and voltage-dependent gating variable  variable  ACh- and voltage-dependent gating  variable of |
| 29  9 | *[cAMP]* | 19.73 | pmol/mg protein | cAMP |
| 30  9 | *[PLB]* | 0.23 | [] | PLB phosphorylation level |
| 31 | *A* | 0.06 | [] | The density of regulatory units with bound Ca^2+^ and adjacent weak cross-bridges |
| 32 | *TT* | 0.02 | [] | The density of regulatory units with bound Ca^2+^ and adjacent strong cross-bridge |
| 33 | *U* | 0.06 | [] | The density of regulatory units without bound but with adjacent strong cross-bridge |
| 34 | *SL* | 1.75 | µm | Sarcomere length |

## S.2 Model constants

| **Symbol** | **Value** | **Units** | **Description** |
| --- | --- | --- | --- |
| **Mitochondrial Ca^2+^ parameters within the numerical model** | | | |
| ${{[Ca}^{2+}]}_{o}$ | 2 | mM | Extracellular Ca^2+^ |
| ${{[K}^{+}]}_{o}$ | 5.4 | mM | Extracellular K^+^ |
| ${{[K}^{+}]}_{i}$ | 140 | mM | Intracellular K^+^ |
| ${{[Na}^{+}]}_{o}$ | 140 | mM | Extracellular Na^+^ |
| ${{[Na}^{+}]}_{i}$ | 10 | mM | Intracellular Na^+^ |
| ${{[Mg}^{+}]}_{i}$ | 2.5 | mM | Intracellular Mg^2+^ |
| ${ATP}_{m}$ | 2.533 | mM | Intracellular ATP |
| $E_{M,protein}$ | 600 | [] | Conversion from mM to pmol/mg protein |
| **Cell compartments within the numerical model** | | | |
| $C_{m}$ | 32 | pF | Cell electric capacitance |
| $V_{cell}$ | 3.5185838 | pL | Cell volume |
| $V_{sub}$ | 0.035097874 | pL | Submembrane space volume |
| $V_{jSR}$ | 0.0042 | pL | Volume of junctional SR (Ca^2+^ release store) |
| $V_{i}$ | 1.5835 | pL | Myoplasmic volume |
| $V_{nSR}$ | 0.0408 | pL | Volume of network SR (Ca^2+^ uptake store) |
| $V_{myto}$ | 0.6334 | pL | Mitochondrial volume |
| *F* | 96485 | C/M | Faraday constant |
| *T* | 310.15 | K˚ | Absolute temperature for 37˚C |
| *R* | 8.3144 | J/(M·K˚) | Universal gas constant |
| $E_{T}$ | 26.72 | mV | RT/F |
| $E_{CaL}$ | 45 | mV | Apparent reversal potential of *I_CaL_* |
| $E_{CaT}$ | 45 | mV | Apparent reversal potential of *I_CaT_* |
| $E_{st}$ | 37.4 | mV | Apparent reversal potential of *I_st_* |
| **AC-cAMP/PKA signaling parameters within the numerical model** | | | |
| $K_{AC,I}$ | 0.016 | 1/min | Non- Ca^2+^ AC activity |
| $K_{AC}$ | 0.0735 | 1/min | Non-Ca^2+^ AC activation |
| $K_{Ca}$ | 0.000178 | mM | Maximal Ca^2+^ AC activation |
| $K_{AC,Ca}$ | 0.000024 | mM | Half-maximal Ca^2+^ AC activation |
| $k_{PKA}$ | 9000 | pmol/protein/min | Maximal PKA activity |
| $k_{PKA,cAMP}$ | 284.5 | pmol/protein | Half-maximal PKA activation |
| $n_{PKA}$ | 5 | [] | Hill coefficient |

| **Symbol** | **Value** | **Units** | **Description** |
| --- | --- | --- | --- |
| **Phosphorylation parameters** | | | |
| $k_{PLBp}$ | 52.25 | 1/min | Maximal PLB phosphorylation |
| $n_{PLB}$ | 1 | [] | Hill coefficient |
| $k_{PKA,PLB}$ | 1.651 | [] | Half-maximal PLB phosphorylation |
| *[PP1]* | 0.89 | µM | PP1 concentration |
| $k_{PP1}$ | 23.575 | 1/µM/min | Maximal PP1 activity |
| $K_{PP1,PLB}$ | 0.06967 | [] | Half-maximal PP1 activity |
| **Membrane parameters** | | | |
| $g_{CaL,max}$ | 0.9 | nS/pF | Maximal L-type Ca^2+^ current conductance |
| $K_{mfCa}$ | 0.00035 | mM | Dissociation constant of Ca^2+^ -dependent *I_CaL_* inactivation |
| $\alpha_{fCa}$ | 0.021 | ms^-1^ | Ca^2+^ dissociation rate constant for *I_CaL_* |
| $g_{CaT,max}$ | 0.1832 | nS/pF | Maximal T-type Ca^2+^ current conductance |
| $g_{bCa,max}$ | 0.0006 | nS/pF | Maximal background Ca^2+^ current conductance |
| $g_{If,max}$ | 0.1 | nS/pF | Maximal hyperpolarization activated current conductance |
| $K_{if}$ | 24.4 | mV | Maximal *I_f_* activation by cAMP |
| $K_{0.5,if}$ | 17.57 | pmol/mg protein | Half-maximal *I_f_* activation by cAMP |
| $n_{if}$ | 9.281 | [] | Hill coefficient |
| $V_{I_{f},0.5}$ | -64 | mV | Half activation voltage of the funny current in the basal state |
| $g_{st,max}$ | 0.00001 | nS/pF | Maximal sustained inward current conductance |
| $g_{Kr,max}$ | 0.1 | nS/pF | Maximal delayed rectifier K^+^ current rapid component conductance |
| $g_{Ks,max}$ | 0.013 | nS/pF | Maximal delayed rectifier K^+^ current slow component conductance |
| $g_{to,max}$ | 0.252 | nS/pF | Maximal 4-aminopyridine sensitive transient K^+^ current conductance |
| $g_{sus,max}$ | 0.02 | nS/pF | Maximal 4-aminopyridine sensitive sustained K^+^ current conductance |
| $g_{NaK,max}$ | 2.88 | pA/pF | Maximal Na^+^/K^+^ pump current conductance |
| $K_{mKp}$ | 1.4 | mM | Half-maximal *K*_o_ for *I_NaK_* |
| $K_{mNap}$ | 14 | mM | Half-maximal *Na*_i_ for *I_NaK_* |
| $g_{bNa}$ | 0.00486 | nS/pF | Maximal background Na^+^ current conductance |
| $g_{ACh}$ | 0.31332 | nS/pF | Maximal acetylcholine-activated K^+^ current |
| **Na^+^/Ca^2+^ exchanger current parameters** | | | |
| $k_{NCX}$ | 225 | pA/pF | Maximal Na^+^/ Ca^2+^ exchanger current conductance |
| $K_{1ni}$ | 395.3 | [] | Intracellular Na^+^ binding to first site on NCX |
| $K_{2ni}$ | 2.289 | [] | Intracellular Na^+^ binding to second site on NCX |
| $K_{3ni}$ | 26.44 | [] | Intracellular Na^+^ binding to third site on NCX |
| $K_{1no}$ | 1628 | [] | Extracellular Na^+^ binding to first site on NCX |
| $K_{2no}$ | 561.4 | [] | Extracellular Na^+^ binding to second site on NCX |
| $K_{3no}$ | 4.663 | [] | Extracellular Na^+^ binding to third site on NCX |
| $K_{ci}$ | 0.0207 | [] | Intracellular Ca^2+^ binding to NCX transporter |
| $K_{co}$ | 3.663 | [] | Extracellular Ca^2+^ binding to NCX transporter |
| $K_{cni}$ | 26.44 | [] | Intracellular Na^+^ and Ca^2+^ simultaneous binding to NCX |
| $Q_{ci}$ | 0.1369 | [] | Intracellular Ca^2+^ occlusion reaction of NCX |
| $Q_{co}$ | 0 | [] | Extracellular Ca^2+^ occlusion reaction of NCX |
| $Q_{n}$ | 0.4315 | [] | Na^+^ occlusion reactions of NCX |

| **Ca^2+^ flux parameters** | | | |
| --- | --- | --- | --- |
| $\tau_{diff,Ca}$ | 0.04 | ms | Time constant of Ca^2+^ diffusion from the submembrane to myoplasm |
| $\tau_{tr}$ | 40 | ms | Time constant for Ca^2+^ transfer from the network to junctional SR |
| $K_{up}$ | 0.6·10^-3^ | mM | Half-maximal Ca_i_ for Ca^2+^ uptake in the network SR |
| $P_{up,basal}$ | 0.0096 | mM /ms | Rate constant for Ca^2+^ uptake by the Ca^2+^ pump in the network SR |
| $k_{oCa,max}$ | 10 | mM ^-2^· ms^-1^ | Ryanodine channel parameters |
| ${RyR}_{min}$ | 0.0127 | [] |  |
| ${RyR}_{max}$ | 0.02 | [] |  |
| $k_{0.5,RyR}$ | 0.7 | [] |  |
| $n_{RyR}$ | 9.773 | [] |  |
| $k_{om}$ | 0.06 | ms^-1^ |  |
| $k_{iCa}$ | 0.5 | mM ^-1^· ms^-1^ |  |
| $k_{im}$ | 0.005 | ms^-1^ |  |
| ${EC}_{50\_SR}$ | 0.45 | mM |  |
| $k_{5}$ | 400·10^3^ | ms^-1^ |  |
| ${Max}_{SR}$ | 13 | [] |  |
| ${Min}_{SR}$ | 1 | [] |  |
| *HSR* | 3 | [] |  |
| **Natural buffering of Ca^2+^ and Mg^2+^parameters** | | | |
| $k_{bCM}$ | 0.542 | ms^-1^ | Ca^2+^ dissociation constant for calmodulin |
| $k_{bCQ}$ | 0.445 | ms^-1^ | Ca^2+^ dissociation constant for calsequestrin |
| $k_{fCM}$ | 227.7 | mM ^-1^· ms^-1^ | Ca^2+^ association constant for calmodulin |
| $k_{fCQ}$ | 0.534 | mM ^-1^· ms^-1^ | Ca^2+^ association constant for calsequestrin |
| ${TC}_{tot}$ | 0.042 | mM | Total concentration of the troponin- Ca^2+^ site |
| ${CQ}_{tot}$ | 10 | mM | Total calsequestrin concentration |
| ${CM}_{tot}$ | 0.045 | mM | Total calmodulin concentration |

| **Symbol** | **Value** | **Units** | **Description** |
| --- | --- | --- | --- |
| **Mitochondrial Ca^2+^ parameters** | | | |
| $\beta_{Ca}$ | 0.1 | [] | The fraction of Ca^2+^ that binds to Ca^2+^ buffers in the mitochondria |
| $P_{Ca}$ | 0.256 | ms^-1^ | Uniporter Ca^2+^ permeability |
| $\Psi_{m}$ | 154.226 | mV | Mitochondrial membrane potential |
| $\alpha_{m}$ | 0.2 | [] | Mitochondrial activity coefficients |
| $\alpha_{e}$ | 0.341 | [] | Extra mitochondrial activity coefficients |
| $K_{Ca,m}$ | 0.003 | mM | Na^+^-Ca^2+^ exchanger Ca^2+^ affinity |
| $z_{Ca}$ | 2 | [] |  |
| **Force parameters** | | | |
| ${SL}_{0}$ | 0.8 | µm | A constant coefﬁcient that describes the effect of the actin- and myosin-ﬁlament lengths on the single overlap length. Actin- and myosin-ﬁlament lengths on the single overlap length |
| $N_{c}$ | 2*10^13^ | 1/mm^2^ | The SAN cross-section area |
| $F_{k0}$ | 350 | 1/mM | The cross-bridge independent coefﬁcient of calcium  afﬁnity. |
| $F_{k1}$ | 3000 | 1/mM | The cooperativity coefﬁcient. Describes the dependence of calcium afﬁnity on the number of strong cross-bridges |
| *FN* | 3.5 |  | Hill coefficient |
| $F_{k,0.5}$ | 2.5*10^9^ | 1/mm^3^ | Half-maximal cross-bridge Ca^2+^ affinity |
| $F_{kl}$ | 60 | 1/mM/ms | The rate constant of calcium binding to troponin  low-afﬁnity sites. |
| $F_{f}$ | 0.04 | 1/ms | The cross-bridge turnover rate from the weak to the strong conformation. |
| $F_{g0}$ | 0.03 | 1/ms | The cross-bridge weakening rate at isometric regime |
| $F_{gl}$ | 4.4*10^6^ | 1/m | The mechanical-feedback coefﬁcient. Describes the dependence of the XB weakening rate on the shortening velocity |
| $F_{xb}$ | 2*10^-9^ | mN | The unitary force per cross-bridge at isometric regime |
| **ATP parameters** | | | |
| ${ATP}_{i,max}$ | 0.02533 | mM |  |
| $k_{ATP}$ | 61420 | [] |  |
| $k_{ATP,0.5}$ | 6724 | [] |  |
| *cAMPb* | 20 | pmol/ mg protein | Baseline cAMP concentration |
| $n_{ATP}$ | 3.36 | [] |  |
| $K_{ATP,min}$ | 6034 | [] |  |
| **Cage experiments** | | | |
| $k_{cAMP,on}$ | 10^-5^ | mM^-1^ ms^-1^ | Rate of cAMP caging |
| $k_{cAMP,off}$ | 0.3 | ms^-1^ | Rate of cAMP release from the cage |
| $k_{ISO,on}$ | 10^-7^ | mM^-1^ ms^-1^ | Rate of ISO caging |
| $k_{ISO,off}$ | 0.11 | ms^-1^ | Rate of ISO release from the cage |

## S.3 Equations

### S.3.1 General

Fig 1 shows a schematic illustration of the sinoatrial node mechanisms. Fig S1 shows the autonomic regulation of key protein targets on the M and Ca^2+^ clocks via -AC-cAMP-PKA signaling. It is assumed that the net current passing through the membrane is determined by the following components:

$$I=I_{CaL}+I_{CaT}+I_{bCa}+I_{f}+I_{st}+I_{Kr}+I_{Ks}+I_{NaK}+I_{NCX}+I_{bNa}+I_{to}+I_{sus}+I_{KACh},$$

where $I$ is the sum of all membrane currents, $I_{CaL}$the L- type Ca^2+^ current,$I_{CaT}$ the T-type Ca^2+^ current, $I_{bCa}$ the Ca^2+^ background current, $I_{f}$ the hyperpolarization- activated funny current, $I_{st}$ the sustained inward current, $I_{Kr}$ the rapidly activating delayed rectifier K^+^ current, $I_{Ks}$ the slow activating delayed rectifier K^+^ current, $I_{NaK}$the Na^+^ -K^+^ pump current, $I_{NCX}$ the Na^+^-Ca^2+^ exchanger current, $I_{bNa}$ the Na^+^ dependent current, $I_{to}$ and $I_{sus}$ the 4-aminopyridine- sensitive currents, and $I_{KACh}$the ACh activated muscarinic current. The rate of change of the membrane potential can be obtained using the following equation:

$$\frac{dV_{m}}{dt}=-\left( \frac{1}{C_{m}} \right)\times I,$$

where $C_{m}$ is the membrane capacitance and $V_{m}$ is the membrane potential.

### S.3.2 AC-cAMP PKA signaling

### S.3.2.1 cAMP activity

The expressions for k_1_ and k_3_ were taken from (12). The expressions for k_iso_ and k_cch_ were evaluated using the experimental data from this contribution together with experimental results from (20). The expression for k_2_ (PDE inhibition of cAMP) was assumed to follow a Hill-like equation, so that at a high [cAMP] level, the amount of PDE saturates, i.e., there is a defined quantity of PDE in a cell that can degrade a maximal amount of [cAMP]. In addition, as we approach [cAMP]/[cAMP]_basal_ = 50%, the amount of PDE that inhibits cAMP is assumed to tending toward zero, as for this concentration of cAMP, the AP rate tends toward zero (20). Fig S2 shows the curve of k­_iso_ and k_cch_. Also refer to Fig 2 for a schematic illustration of the AC-cAMP-PKA signaling cascade. The division by 6000 converts the units from min^-1^ to ms^-1^.

$$\frac{d[cAMP]}{dt}=\left( (k_{iso}-k_{cch})\cdot\left[ ATP \right]+k_{1}\cdot\left[ ATP \right]-k_{2}\cdot\left[ cAMP \right]- k_{3}\cdot\left[ cAMP \right] \right)/6000,$$

$$k_{iso}=0.0070+0.1181 \cdot\frac{{[ISO]}^{0.8664}}{{48.1212}^{0.8664}+{[ISO]}^{0.8664}}$$

$$k_{cch}=0.0146\cdot\frac{{[CCh]}^{1.4402}}{{51.7331}^{1.4402}+{[CCh]}^{1.4402}}$$

$$k_{1}=K_{AC,I}+\frac{K_{AC}}{1+\exp\left( {(K}_{Ca}-k_{bCM}\cdot\frac{f_{CMi}}{k_{fCM}\cdot\left( 1-f_{CMi} \right)})/K_{AC,Ca} \right)}$$

$$k_{2}=1.147\cdot240.0805\cdot\frac{\left[ cAMP \right]^{1.9921}}{{20.97}^{2.9921}+\left[ cAMP \right]^{2.9921}}$$

$$k_{3}=k_{PKA}\cdot\frac{\left[ cAMP \right]^{{(n}_{PKA}-1)}}{k_{PKA,cAMP}^{n_{PKA}}+\left[ cAMP \right]^{n_{PKA}}}.$$

### S.3.2.2 PLB activity

The expressions for k_4_ and k_5_ were taken from (1). These functions were obtained by curve fitting using some experimental data from (2, 3). Also refer to Fig 2 for a schematic illustration of the PLB_p_ production and degradation kinetic. The division by 6000 converts the units from min^-1^ to ms^-1^.

$$\frac{d[{PLB}_{p}]}{dt}=\left( k_{4}\cdot\left[ PKA \right]-k_{5}\cdot\left[ {PLB}_{p} \right] \right)/6000,$$

$$k_{4}={(k}_{{PLB}_{p}}\cdot\left[ PKA \right]^{n_{PLB}-1})/(k_{PKA,PLB}^{n_{PLB}}+{[PKA]}^{n_{PLB}})$$

$$k_{5}=k_{PP1}\cdot\frac{\left[ PP1 \right]}{k_{PP1,PLB}+\left[ {PLB}_{p} \right]}.$$

### S.3.2.3 PKA activity

The explicit cAMP-PKA relationship was adapted from the Saucerman *et al.* model (4). The system of equations was solved algebraically (see plot Fig S3).

$$\left[ PKA \right]=2-\left[ RC \right]-\left[ ARC \right]-\left[ A_{2}RC \right]-\left[ PKA_{PKI} \right]$$

$$\left[ A_{2}R \right]=\left[ PKA \right]+\left[ PKA_{PKI} \right]$$

$$\left[ A_{2}RC \right]=\left[ PKA \right]\cdot[A_{2}R]/0.009$$

$$\left[ ARC \right]=0.008\cdot\left[ A_{2}RC \right]/[cAMP]$$

$$\left[ PKA_{PKI} \right]=\left[ {PKI}_{tot} \right]\cdot[PKA]/(0.001+[PKA] )$$

$$\left[ RC \right]=0.008\cdot\frac{\left[ A_{2}RC \right]}{\left[ cAMP \right]}.$$

### S.3.3 Membrane currents

### S.3.3.1 L-type Ca^2+^ current, *I_CaL_*

The equations were taken from Maltsev and Lakatta (5), who themselves adapted them from the model by Kurata *et al.* (6) (the expressions of the equations are the same as in the Kurata model with some minor modification of some of the constants). The modulation of *I_CaL_* by PKA was created by using the experimental measurement from Lyashkov et al. (7), who measured a maximal inactivation of *I_CaL_* of 20%, and Vinogradova *et al.* (8), who measured a maximal activation of +80%; see Fig S4 for the curve fitting of b_CaL_.

$$I_{CaL}= C_{m}\cdot g_{CaLmax}\cdot(1+b_{CaL})\cdot(V_{m}-E_{CaL})\cdot d_{L}\cdot f_{L}\cdot f_{Ca}$$

with

$$b_{CaL}=-0.2152+1.6913\cdot{PKA}^{10.0808}/({0.8836}^{10.0808}+{PKA}^{10.0808})$$

$$d_{L,\infty}={\{1+exp(-(V_{m}+13.5)/6\}}^{-1}$$

$$f_{L,\infty}={\{1+exp((V_{m}+35)/7.3\}}^{-1}$$

$$f_{Ca,\infty}=K_{mfCa}/(K_{mfCa}+{[{Ca}^{2+}]}_{sub})$$

$$\alpha_{dL}=\frac{-0.02839\cdot(V_{m}+35)}{\exp\left( -\frac{V_{m}+35}{2.5} \right)-1}-\frac{0.0849\cdot V_{m}}{\exp\left( -\frac{V_{m}}{4.8} \right)-1}$$

$$\beta_{dL}=0.01143\cdot(V_{m}-5)/\{exp((V_{m}-5)/2.5)-1\}$$

$$\tau_{dL}={\{\alpha_{dL}+\beta_{dL}\}}^{-1}$$

$$\tau_{fL}=257.1\cdot\exp\left( -{[{(V}_{m}+32.5)/13.9]}^{2} \right)+44.3$$

$$\tau_{fCa}=\frac{f_{Ca,\infty}}{\alpha_{fCa}}.$$

### S.3.3.2 Hyperpolarization activated “funny” current, *I_f_*

The model for the funny current is based on (1, 6). The shift in the I-V curve is described by V_shift_ and plotted in Fig 12. Data from (9, 10) were used to do the curve fitting. Based on (10): MDL (10 µM) shifts the activation curve by -17 mV; IBMX, 100 µM shifts the current-activation curve by 7.2 mV; BAPTA 5 µM leads to a -10 mV shift. 1µ of ISO shifted the current-activation curve by 7.2 mV (9). In addition, zero shift was assumed for the basal cAMP value (i.e. [cAMP] = 20 pmol/mg proteins). Fig S5 shows the value of V_shift_ as a function of [cAMP].

$$I_{f}=I_{fNa}+I_{fK}$$

with

$$I_{fNa}=C_{m}\cdot0.3833\cdot g_{I_{f,max}}\cdot\left( V_{m}-E_{Na} \right)\cdot y^{2}$$

$$I_{fK}=C_{m}\cdot0.6167\cdot g_{I_{f,max}}\cdot\left( V_{m}-E_{K} \right)\cdot y^{2}$$

$$V_{shift}=K_{if}\cdot\frac{\left[ cAMP \right]^{n_{if}}}{\left\{ K_{0.5,if}^{n_{if}}+\left[ cAMP \right]^{n_{if}} \right\}}-18.76$$

$$y_{\infty}=1/(1+exp((V_{m}-V_{shift}-V_{I_{f},0.5})/13.5))$$

$$\tau_{y}=0.7166529/\{exp((-(V_{m}+386.9)/45.302)+exp((V_{m}-73.08)/19.231))\}$$

$$E_{Na}=E_{T}\cdot ln({{[Na}^{+}]}_{0}/{{[Na}^{+}]}_{i})$$

$$E_{K}=E_{T}\cdot\ln\left( \frac{{{[K}^{+}]}_{0}}{{{[K}^{+}]}_{i}} \right).$$

### S.3.3.3 T-type Ca^2+^ current, *I_CaT_*

The model is based on Demir *et al.* (11) and was modified using the model of Kurata *et al.* (6).

$$I_{CaT}=C_{m}\cdot g_{CaT,max}\cdot(V_{m}-E_{CaT})\cdot d_{T}\cdot f_{T}$$

with

$$d_{T,\infty}=1/(1+exp(-(V_{m}+26.3)/6))$$

$$f_{T,\infty}=1/(1+exp(V_{m}+61.7)/5.6)$$

$$\tau_{dT}=1/[1.068\cdot exp((V_{m}+26.3)/30)+1.068\cdot exp(-(V_{m}+26.3)/30)]$$

$$\tau_{fT}=1/[0.0153\cdot exp(-(V_{m}+61.7)/83.3)+0.015\cdot exp((V_{m}+61.7)/15.38)].$$

### S.3.3.4 Rapidly activating delayed rectifier K^+^ current, *I_Kr_*

The model from Kurata *et al.* was used (6).

$$I_{Kr}=C_{m}\cdot g_{Kr,max}\cdot(V_{m}-E_{K})\cdot(0.6\cdot p_{aF}+0.4\cdot p_{aS})\cdot p_{i}$$

with

$$E_{K}=E_{T}\cdot ln(K_{o}/K_{i})$$

$$p_{aF,\infty}=p_{aS,\infty}=1/[1+exp(-(V_{m}+23.2)/10.6)]$$

$$p_{i,\infty}=1/[1+exp((V_{m}+28.6)/17.1)]$$

$$\tau_{paF}=0.84655354/[0.0372\cdot exp(V_{m}/15.9)+0.00096\cdot exp(-(V_{m}/22.5))]$$

$$\tau_{paS}=0.84655354/[0.0042\cdot exp(V_{m}/17)+0.00015\cdot exp(-(V_{m}/21.6))$$

$$\tau_{pi}=1/[0.1\cdot exp(-V_{m}/54.645)+0.656\cdot exp(V_{m}/106.157)].$$

### S.3.3.5 Slow activating delayed rectifier K^+^ current, *I_Ks_*

The channel current formulation is based on the Zhang et al. model (12).

$$I_{Ks}=C_{m}\cdot g_{Ks}\cdot(V_{m}-E_{Ks})\cdot n^{2}$$

with

$$E_{Ks}=E_{T}\cdot ln({(K}_{0}+0.12\cdot\left[ {Na}^{+} \right]_{0})/{(K}_{i}+0.12\cdot\left[ {Na}^{+} \right]_{i}))$$

$$\alpha_{n}=0.014/(1+exp(-(V_{m}-40)/9))$$

$$\beta_{n}=0.001\cdot exp(-V_{m}/45)$$

$$n_{\infty}=\alpha_{n}/(\alpha_{n}+\beta_{n})$$

$$\tau_{n}=\frac{1}{\alpha_{n}+\beta_{n}}.$$

### S.3.3.6 4-aminopyridine-sensitive currents, *I*_4AP_ = *I*_to_ *+ I_sus_*

The channel current formulation is based on the Zhang *et al.* model (12).

$$I_{to}=C_{m}\cdot g_{to,max}\cdot(V_{m}-E_{K})\cdot q\cdot r$$

$$I_{sus}=C_{m}\cdot g_{sus,max}\cdot(V_{m}-E_{K}) \cdot r$$

with

$$q_{\infty}=1/[1+exp({(V}_{m}+49)/13)]$$

$$r_{\infty}=1/[1+exp(-{(V}_{m}-19.3)/15)]$$

$$\tau_{q}=39.102/[0.57\cdot\exp\left( -0.08\cdot\left( V_{m}+44 \right) \right)+0.065\cdot exp(0.1\cdot(V_{m}+45.93))]+6.06$$

$$\tau_{r}=14.40516/[1.037\cdot\exp\left( 0.09\cdot\left( V_{m}+30.61 \right) \right)+0.369\cdot exp(-0.12\cdot(V_{m}+23.84))]+2.75352.$$

### S.3.3.7 Sustained inward current, *I_st_*

The formulation from Kurata et *al.* was used (6).

$$I_{st}=C_{m}\cdot g_{st,max}\cdot{(V}_{m}-E_{st})\cdot q_{a}\cdot q_{i}$$

with

$$q_{a,\infty}=1/[1+exp(-{(V}_{m}+57)/5)]$$

$$\alpha_{qa}=1/[0.15\cdot exp(-{(V}_{m}/11))+0.2\cdot exp(-V_{m}/700)]$$

$$\beta_{qa}=1/[16\cdot\exp{(V}_{m}/8)+15\cdot\exp{(V}_{m}/50)]$$

$$\tau_{qa}=1/(\alpha_{qa}+\beta_{qa})$$

$$\alpha_{qi}=1/[3100\cdot exp(-{(V}_{m}/13))+700\cdot exp(V_{m}/70)]$$

$$\beta_{qi}=1/[95\cdot exp(-{(V}_{m}/10))+50\cdot exp(-{(V}_{m}/700))]+0.000229/[1+exp(-V_{m}/5)]$$

$$\tau_{qi}=6.65/(\alpha_{qi}+\beta_{qi})$$

$$q_{i,\infty}=\alpha_{qi}/(\alpha_{qi}+\beta_{qi}).$$

### S.3.3.8 Na^+^-dependent background current, *I_bNa_*

The Kurata *et al.* (6) model was used. The conductance parameter was updated as in (5).

$$I_{b,Na}=C_{m}\cdot g_{bNa}\cdot{(V}_{m}-E_{Na}).$$

### S.3.3.9 Na^+^-K^+^ pump current, *I_NaK_*

The formulation from Kurata *et al.* was used (6).

$$I_{NaK}=C_{m}\cdot\frac{I_{NaK,max}}{1+\left( \frac{K_{mKp}}{K_{0}} \right)^{1.2}}\cdot\frac{1}{1+\left( \frac{K_{mNap}}{\left[ {Na}^{+} \right]_{i}} \right)^{1.3}}\cdot\frac{1}{1+\exp\left( -{(V}_{m}-E_{Na}+120 \right)/30)}.$$

### S.3.3.10 Ca^2+^- background current, *I_bCa_*

The formulation and parameter values in (5) were used.

$$I_{bCa}=C_{m}\cdot g_{bCa}\cdot\left( V_{m}-E_{CaL} \right).$$

### S.3.3.11 Acetylcholine-activated K^+^ current, *I_KACh_*

The formulation of the current is based on the original work of (13) and its further modification in (14):

$$I_{KACh}=C_{m}\cdot g_{KACh}\cdot\left( V_{m}-E_{K} \right)$$

$$\beta_{w}=0.001\cdot12.32/(1+0.0042/([CCh]\cdot{10}^{-6}))$$

$$\alpha_{w}=0.001\cdot17\cdot exp(0.0133\cdot(V_{m}+40))$$

$$w_{\infty}=\beta_{w}/(\alpha_{w}+\beta_{w})$$

$$\tau_{w}=1/(\alpha_{w}+\beta_{w})$$

### S.3.3.12 Na^+^-Ca^2+^ exchanger current, *I_NCX_*

The formulation of the *I_NCX_* current is based on Dokos *et al.* (15).

$$I_{NCX}=C_{m}\cdot k_{NCX}\cdot\left[ \frac{k_{21}\cdot x_{2}-k_{12}\cdot x_{1}}{x_{1}+x_{2}+x_{3}+x_{4}} \right].$$

$$d_{0}=1+\left( \frac{{{[Ca}^{2+}]}_{0}}{K_{co}} \right)\cdot(1+exp(Q\_co\cdot V_{m}/E_{T} ))+({{[Na}^{+}]}_{0}/K_{1no})\cdot\{1+({{[Na}^{+}]}_{0}/K_{2no})\cdot(1+{{[Na}^{+}]}_{0}/K_{3no})\}$$

$$k_{43}={{[Na}^{+}]}_{i}/(K_{3ni}+{{[Na}^{+}]}_{i})$$

$$k_{41}=exp[-Q_{n}\cdot V_{m}/(2\cdot E_{T})]$$

$$k_{34}={{[Na}^{+}]}_{0}/(K_{3no}+{{[Na}^{+}]}_{o})$$

$$k_{21}=({{[Ca}^{2+}]}_{0}/K_{co})\cdot exp(Q_{CO}\cdot V_{m}/E_{T})/d_{0}$$

$$k_{23}=({{[Na}^{+}]}_{0}/K_{1no})\cdot({{[Na}^{+}]}_{0}/K_{2no})\cdot(1+{{[Na}^{+}]}_{0}/K_{3no})\cdot exp(-Q_{n}\cdot V_{m}/(2\cdot E_{T}))/d_{0}$$

$$k_{32}=exp[Q_{n}\cdot V_{m}/(2\cdot E_{T})]$$

$$x_{1}=k_{34}\cdot k_{41}\cdot\left( k_{23}+k_{21} \right)+k_{21}\cdot k_{32}\cdot(k_{43}+k_{41})$$

$$d_{i}=1+({{[Ca}^{2+}]}_{sub}/K_{ci})\cdot\{1+exp(-Q_{Ci}\cdot V_{m}/E_{T})+{{[Na}^{+}]}_{i}/K_{cni}\}+({{[Na}^{+}]}_{i}/K_{1ni})\cdot\{1+({{[Na}^{+}]}_{i}/K_{2ni})\cdot(1+{{[Na}^{+}]}_{i}/K_{3ni})$$

$$k_{12}={{([Ca}^{2+}]}_{sub}/K_{Ci}) \cdot exp(-Q_{Ci}\cdot V_{m}/E_{T})/d_{i}$$

$$k_{14}=({{[Na}^{+}]}_{i}/K_{1ni})\cdot({{[Na}^{+}]}_{i}/K_{2ni})\cdot(1+{{[Na}^{+}]}_{i}/K_{3ni})\cdot exp(Q_{n}\cdot V_{m}/(2\cdot E_{T}))/d_{i}$$

$$x_{2}=k_{43}\cdot k_{32}\cdot\left( k_{14}+k_{12} \right)+k_{41}\cdot k_{12}\cdot(k_{34}+k_{32})$$

$$x_{3}=k_{43}\cdot k_{14}\cdot\left( k_{23}+k_{21} \right)+k_{12}\cdot k_{23}\cdot(k_{43}+k_{41})$$

$$x_{4}=k_{34}\cdot k_{23}\cdot\left( k_{14}+k_{12} \right)+k_{21}\cdot k_{14}\cdot\left( k_{34}+k_{32} \right).$$

### S.3.4 Sarcoplasmic Reticulum Ca^2+^ cycling

### S.3.4.1 RyR function

The formulation of RyR is based on the work of (16, 17). This RyR modeling was first used in the context of SANC modeling in Maltsev *et al*. (5). The RyR modulation by PKA was taken from Yaniv *et al*. (1).

$$j_{SRCarel}=k_{s}\cdot O\cdot({{[Ca}^{2+}]}_{jSR}-{{[Ca}^{2+}]}_{sub})$$

with

$$k_{CaSR}={Max}_{SR}-({Max}_{SR}-{Min}_{SR})/(1+{({EC}_{50,SR}/{{[Ca}^{2+}]}_{jSR})}^{HSR})$$

$$k_{oCa}=k_{{oCa}_{max}}\cdot({RyR}_{min}+1-{RyR}_{max}\cdot\left[ PKA \right]^{n_{RyR}}/(k_{0.5,RyR}^{n_{RyR}}+{[PKA]}^{n_{RyR}}))$$

$$k_{oSRCa}=k_{oCa}/k_{CaSR}$$

$$k_{iSRCa}=k_{iCa}\cdot k_{CaSR}$$

$$dR/dt={(k}_{im} \cdot RI-k_{iSRCa}\cdot{{[Ca}^{2+}]}_{sub}\cdot R)-(k_{oSRCa}\cdot{{[Ca}^{2+}]}_{sub}^{2}\cdot R-k_{om}\cdot O)$$

$$dO/dt={(k}_{oSRCa}\cdot{{[Ca}^{2+}]}_{sub}^{2}\cdot R-k_{om}\cdot O)-(k_{iSRCa}\cdot{{[Ca}^{2+}]}_{sub}\cdot O-k_{im}\cdot I)$$

$dI/dt=\left( k_{iSRCa}\cdot{{[Ca}^{2+}]}_{sub}\cdot O-k_{im}\cdot I \right)-(k_{om}\cdot I-k_{oSRCa}\cdot{{[Ca}^{2+}]}_{sub}^{2}\cdot RI)$

$$\frac{dRI}{dt}=\left( k_{om}\cdot I-k_{oSRCa}\cdot{{[Ca}^{2+}]}_{sub}^{2}\cdot RI \right)-\left( k_{im}\cdot RI-k_{iSRCa}\cdot{{[Ca}^{2+}]}_{sub}\cdot R \right).$$

### S.3.4.2 Ca^2+^ diffusion flux from submembrane space to myoplasm, *j_Ca,dif_*

$$j_{Ca,dif}=({{[Ca}^{2+}]}_{sub}-{{[Ca}^{2+}]}_{i})/\tau_{dif,Ca}.$$

**S.3.4.3** **The rate of Ca^2+^ uptake (pumping)** **by the SR, *j_up_***

The formulation of the equation is based on the Rudy *et al.* model (18) and modified to take into account the modulation of the SERCA pump by PLB_p_.

$$j_{up}=P_{up,basal}\cdot F(\left[ {PLB}_{p} \right])/[1+K_{up}/{{[Ca}^{2+}]}_{i}].$$

For $\left[ {PLB}_{p} \right]\leq0.23,$

$$F\left( \left[ {PLB}_{p} \right] \right)=1.698\cdot\left[ {PLB}_{p} \right]^{13.584}/({0.2240}^{13.584}+\left[ {PLB}_{p} \right]^{13.584}).$$

For $\left[ {PLB}_{p} \right]>0.23,$

$$F\left( \left[ {PLB}_{p} \right] \right)=3.3931\cdot\left[ {PLB}_{p} \right]^{4.0695}/{0.2805}^{4.0695}+\left[ {PLB}_{p} \right]^{4.0695}.$$

### S.3.4.4 Ca^2+^ flux between network and junctional SR compartments, *j_tr_*

$$j_{tr}=({{[Ca}^{2+}]}_{nsr}-{{[Ca}^{2+}]}_{jsr})/\tau_{tr}.$$

### S.3.4.5 Natural Ca^2+^ buffering

$$\frac{df_{TC}}{dt}=f_{kl}\cdot{{[Ca}^{2+}]}_{i}\cdot\left( 1-A-TT \right)-k_{l}\cdot(A+TT)$$

$$\frac{df_{CMi}}{dt}=k_{fCM}\cdot{{[Ca}^{2+}]}_{i}\cdot\left( 1-f_{CMi} \right)-k_{bCM}\cdot f_{CMi}$$

$$\frac{df_{CMs}}{dt}=k_{fCM}\cdot{{[Ca}^{2+}]}_{sub}\cdot\left( 1-f_{CMs} \right)-k_{bCM}\cdot f_{CMs}$$

$$\frac{df_{CQ}}{dt}=k_{fCQ}\cdot{{[Ca}^{2+}]}_{jsr}\cdot\left( 1-f_{CQ} \right)-k_{bCQ}\cdot f_{CQ.}$$

See definition for A and TT in the force equations.

### S.3.4.6 Mitochondrial Ca^2+^ fluxes

The uniporter behaves as an ion channel and therefore the flux can be described by the Goldman-Hodgkin-Katz equation, adapted from the Yaniv *et al.* model (1).

$$j_{uni}=P_{Ca}\cdot\frac{z_{Ca}\cdot\Psi_{m}\cdot}{E_{T}}\cdot\frac{\alpha_{m}{{\cdot[Ca}^{2+}]}_{m}\cdot\exp\left[ \frac{-z_{Ca}\cdot\Psi_{m}}{E_{T}} \right]-\alpha_{e}{{\cdot[Ca}^{2+}]}_{i}}{\exp\left[ \frac{-z_{Ca}\cdot\Psi_{m}}{E_{T}} \right]-1}.$$

The Na^+^-Ca^2+^ exchanger flux is described by the Jaffari *et al.* equation (19):

$$j_{NaCa,m}=Q_{mo}\cdot\frac{{{[Ca}^{2+}]}_{m}}{K_{Ca,m}+{{[Ca}^{2+}]}_{m}}$$

### S.3.4.7 Dynamics of Ca^2+^ concentrations in cell compartments

$$\frac{d{{[Ca}^{2+}]}_{i}}{dt}=\frac{j_{Ca,dif}\cdot V_{sub}-j_{up}\cdot V_{nSR}}{V_{i}}-\left( {CM}_{tot}\cdot\frac{df_{{CM}_{i}}}{dt}+{TC}_{tot}\cdot\frac{df_{TC}}{dt} \right)-\frac{\left( j_{uni}-j_{NaCa,m} \right)\cdot V_{myto}}{V_{i}}$$

$$\frac{d{{[Ca}^{2+}]}_{sub}}{dt}=\frac{j_{SRCarel}\cdot V_{jSR}}{V_{sub}}-\frac{1}{2\cdot F\cdot V_{sub}}\cdot\left( I_{CaL}+I_{CaT}+I_{b,Ca}-2\cdot I_{NCX} \right)-(j_{Ca,diff}+{CM}_{tot}\cdot\frac{df_{CMi}}{dt})$$

$$\frac{d{{[Ca}^{2+}]}_{jSR}}{dt}=j_{tr}-j_{SRCarel}-{CQ}_{tot}\cdot\frac{df_{CQ}}{dt}$$

$$\frac{d{{[Ca}^{2+}]}_{nSR}}{dt}=j_{up}-j_{tr}\cdot\frac{V_{jSR}}{V_{nSR}}$$

$$\frac{d{{[Ca}^{2+}]}_{m}}{dt}=(V_{myto}/V_{i})\cdot(j_{uni}-j_{NaCa,m}).$$

### S.3.4.8 Force

The force model parameters are based on the Yaniv-Landesberg model (20) and $N_{c}$ and $F_{XB}$ were fitted based on the experimental results of Catanzaro *et al.* (21).

$$dSL/dt = -V_{e}$$

$$N_{XB}=(SL-{SL}_{0})\cdot N_{c}\cdot(TT-U)\cdot1000/2$$

$$K_{Ca}=F_{ko}+F_{k1}\cdot N_{XB}^{FN}/(F_{k,0.5}^{FN}+N_{XB}^{FN})$$

$$k_{-1}=F_{kl}/K_{Ca}$$

$$dA/dt=F_{kl}\cdot{{[Ca}^{2+}]}_{i}\cdot\left( 1-A-TT-U \right)-A\cdot\left( F_{f}+k_{-l} \right)+TT\cdot(F_{go}-F_{gl}\cdot V_{e})$$

$$dTT/dt=F_{f}\cdot A-TT\cdot\left( F_{g0}+F_{g1}\cdot V_{e}+k_{-l} \right)+F_{kl}\cdot{{[Ca}^{2+}]}_{i}\cdot U$$

$$dU/dt=k_{-l}\cdot TT-(F_{g0}+F_{g1}\cdot V_{e}+F_{kl}\cdot{{[Ca}^{2+}]}_{i}) \cdot U$$

$dV_{e}/dt=0$.

### S.3.4.9 ATP-ADP

The equations were taken from Yaniv *et al.* (1).

$${ATP}_{i}={ATP}_{i,max}\cdot\left( \frac{k_{ATP}\cdot\left( \frac{\left[ cAMP \right]}{cAMPb} \right)^{n_{ATP}}}{k_{ATP,0.5}+\left( \frac{\left[ cAMP \right]}{cAMPb} \right)^{n_{ATP}}}-K_{ATP,min} \right).$$

**S.4 Experiments**

**S.4.1 SANC isolation**

Animals were treated in accordance with the NIH Guide for the Care and Use of Laboratory Animals and the Technion Ethics Committee. The animal protocols have been approved by the Animal Care and Use Committee of the National Institutes of Health (protocol #034LC S2013) and by the Technion (protocol #IL-118-10-13). The rabbits weighed 1.8-2.5 kg and were deeply anaesthetized with sodium pentobarbital (50-90 mg/kg) injected to the central ear vein. The adequacy of anesthesia was monitored until reflexes to ear pinch and jaw tone were lost. The heart was removed quickly and placed on cold PBS buffer. The atrial and the SAN tissues are identified by the physiological marks (superior and inferior vena cava and crista terminalis). These tissue were separated through manual dissection, i.e. the sinoatrial tissue was cut into small strips perpendicular to the crista terminalis and was then dissected away from the atria. For further details how the SANC were isolated see (2).

**S.4.2 Culture Method**

SANC were cultured as previously described (1). In order to measure PKA activity at least 48 h of culture is needed. However, after 48 h in culture the AP firing rate of SANCs decreases (22). To bring the cultured cell function as close as possible to basal function, 1 nM of ISO was used. Using this concentration reach a fresh SANC AP firing rate and phosphorylation activity as suggested in (22). Up to date no other method than culture can be used to infect the SANC with AKAR3. For examples of AP raw data and cell pictures before and after 48h of culture see (1, 22).

**REFERENCES**

1. Yaniv Y, Ganesan A, Yang D, Ziman BD, Lyashkov AE, Levchenko A, et al. Real-time relationship between PKA biochemical signal network dynamics and increased action potential firing rate in heart pacemaker cells: Kinetics of PKA activation in heart pacemaker cells. Journal of Molecular and Cellular Cardiology. 2015;86:168-78.

2. Vinogradova TM, Sirenko S, Lyashkov AE, Younes A, Li Y, Zhu W, et al. Constitutive phosphodiesterase activity restricts spontaneous beating rate of cardiac pacemaker cells by suppressing local Ca2+ releases. Circulation Research. 2008;102(7):761-9.

3. Vinogradova TM, Lyashkov AE, Zhu W, Ruknudin AM, Sirenko S, Yang D, et al. High basal protein kinase A–dependent phosphorylation drives rhythmic internal Ca2+ store oscillations and spontaneous beating of cardiac pacemaker cells. Circulation Research. 2006;98(4):505-14.

4. Saucerman JJ, Brunton LL, Michailova AP, McCulloch AD. Modeling β-adrenergic control of cardiac myocyte contractility in silico. Journal of Biological Chemistry. 2003;278(48):47997-8003.

5. Maltsev VA, Lakatta EG. Synergism of coupled subsarcolemmal Ca2+ clocks and sarcolemmal voltage clocks confers robust and flexible pacemaker function in a novel pacemaker cell model. American Journal of Physiology-Heart and Circulatory Physiology. 2009;296(3):H594-H615.

6. Kurata Y, Hisatome I, Imanishi S, Shibamoto T. Dynamical description of sinoatrial node pacemaking: improved mathematical model for primary pacemaker cell. American Journal of Physiology-Heart and Circulatory Physiology. 2002;283(5):H2074-H101.

7. Lyashkov AE, Vinogradova TM, Zahanich I, Li Y, Younes A, Nuss HB, et al. Cholinergic receptor signaling modulates spontaneous firing of sinoatrial nodal cells via integrated effects on PKA-dependent Ca2+ cycling and IKACh. American Journal of Physiology-Heart and Circulatory Physiology. 2009;297(3):H949-H59.

8. Vinogradova TM, Bogdanov KY, Lakatta EG. β-Adrenergic stimulation modulates ryanodine receptor Ca2+ release during diastolic depolarization to accelerate pacemaker activity in rabbit sinoatrial nodal cells. Circulation Research. 2002;90(1):73-9.

9. Bucchi A, Baruscotti M, Robinson RB, DiFrancesco D. I f-dependent modulation of pacemaker rate mediated by cAMP in the presence of ryanodine in rabbit sino-atrial node cells. Journal of Molecular and Cellular Cardiology. 2003;35(8):905-13.

10. Mattick P, Parrington J, Odia E, Simpson A, Collins T, Terrar D. Ca2+‐stimulated adenylyl cyclase isoform AC1 is preferentially expressed in guinea‐pig sino‐atrial node cells and modulates the If pacemaker current. The Journal of Physiology. 2007;582(3):1195-203.

11. Demir S, Clark J, Murphey C, Giles W. A mathematical model of a rabbit sinoatrial node cell. American Journal of Physiology-Cell Physiology. 1994;266(3):C832-C52.

12. Zhang H, Holden A, Kodama I, Honjo H, Lei M, Varghese T, et al. Mathematical models of action potentials in the periphery and center of the rabbit sinoatrial node. American Journal of Physiology-Heart and Circulatory Physiology. 2000;279(1):H397-H421.

13. Demir SS, Clark JW, Giles WR. Parasympathetic modulation of sinoatrial node pacemaker activity in rabbit heart: a unifying model. American Journal of Physiology-Heart and Circulatory Physiology. 1999;276(6):H2221-H44.

14. Maltsev VA, Lakatta EG. A novel quantitative explanation for the autonomic modulation of cardiac pacemaker cell automaticity via a dynamic system of sarcolemmal and intracellular proteins. American Journal of Physiology-Heart and Circulatory Physiology. 2010;298(6):H2010-H23.

15. Dokos S, Celler B, Lovell N. Ion currents underlying sinoatrial node pacemaker activity: a new single cell mathematical model. Journal of Theoretical Biology. 1996;181(3):245-72.

16. Stern MD, Song LS, Cheng H, Sham JS, Yang HT, Boheler KR, et al. Local control models of cardiac excitation-contraction coupling. A possible role for allosteric interactions between ryanodine receptors. The Journal of general physiology. 1999;113(3):469-89.

17. Shannon TR, Wang F, Puglisi J, Weber C, Bers DM. A mathematical treatment of integrated Ca dynamics within the ventricular myocyte. Biophysical Journal. 2004;87(5):3351-71.

18. Luo C-h, Rudy Y. A dynamic model of the cardiac ventricular action potential. I. Simulations of ionic currents and concentration changes. Circulation Research. 1994;74(6):1071-96.

19. Nguyen M-HT, Dudycha SJ, Jafri MS. Effect of Ca2+ on cardiac mitochondrial energy production is modulated by Na+ and H+ dynamics. American Journal of Physiology-Cell Physiology. 2007;292(6):C2004-C20.

20. Yaniv Y, Sivan R, Landesberg A. Analysis of hystereses in force length and force calcium relations. American Journal of Physiology-Heart and Circulatory Physiology. 2005;288(1):H389-H99.

21. Catanzaro JN, Nett MP, Rota M, Vassalle M. On the mechanisms underlying diastolic voltage oscillations in the sinoatrial node. Journal of Electrocardiology. 2006;39(3):342.

22. Yang D, Lyashkov AE, Li Y, Ziman BD, Lakatta EG. RGS2 overexpression or G i inhibition rescues the impaired PKA signaling and slow AP firing of cultured adult rabbit pacemaker cells. Journal of Molecular and Cellular Cardiology. 2012;53(5):687-94.

**Figure Legends**

**
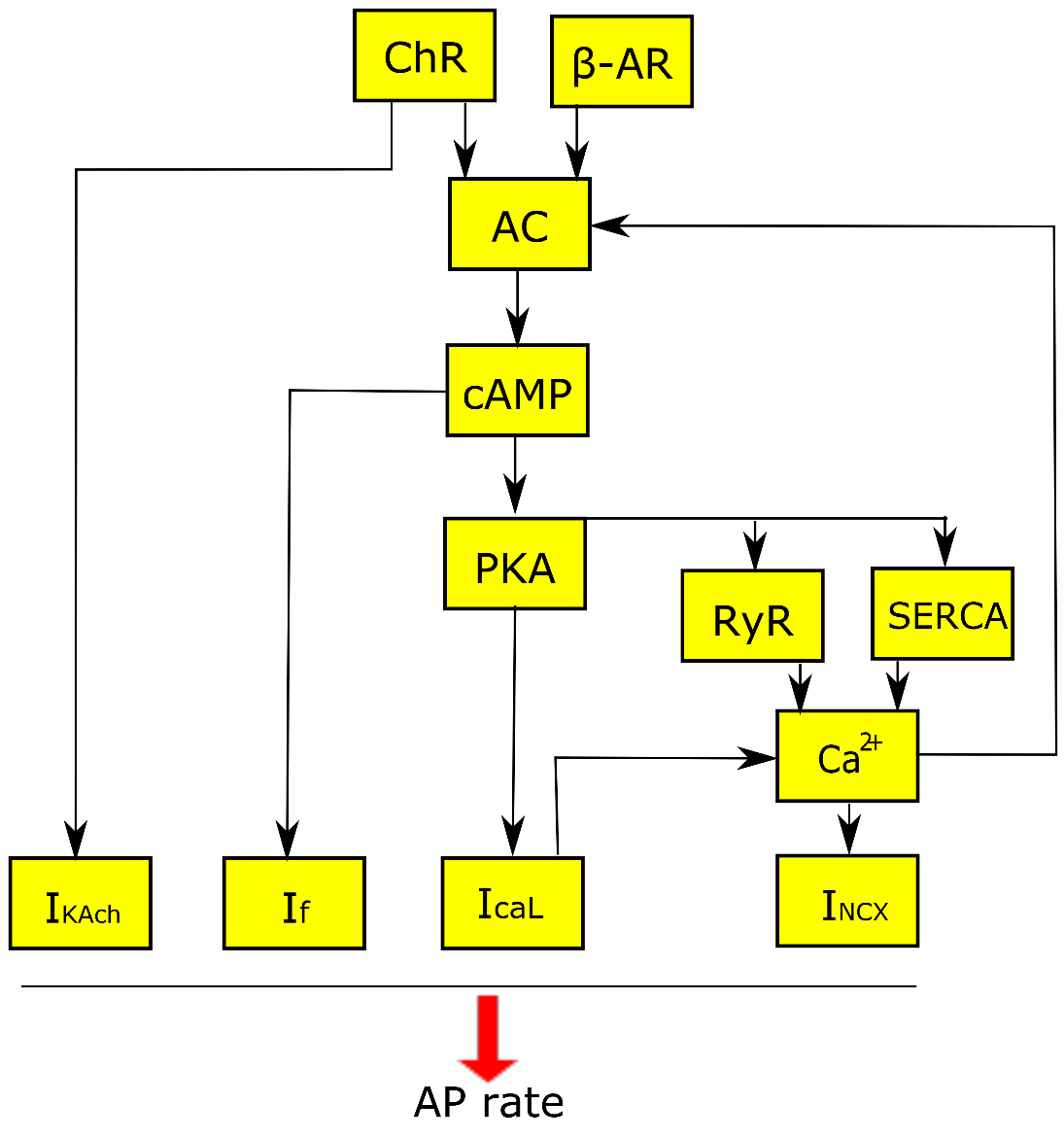
**

**Fig S1:** **Autonomic regulation of key protein targets on the M and Ca^2+^ clocks.** Cholinergic receptor (ChR) and adrenergic receptor (β-AR) stimulation regulate the activation of adenalyse cyclase (AC), which itself regulates the amount of intracellular cAMP and PKA. cAMP shifts the activation curve of the funny current (*I_f_*) and PKA-dependent phosphorylation regulates the activation of the L-type channels (*I_CaL_*) and of proteins located on the sarcoplasmic reticulum: ryanodine (RyR) and the SERCA pump. Thus, the AC-cAMP-PKA signaling affects M and Ca^2+^ clocks function. The activation of these proteins change the intracellular Ca^2+^ concentration, which itself regulates the degree of activated AC via the calmodulin-activated AC. In addition, in the case of ChR stimulation, the muscarinic current (*I_KACh_*) is activated.

**
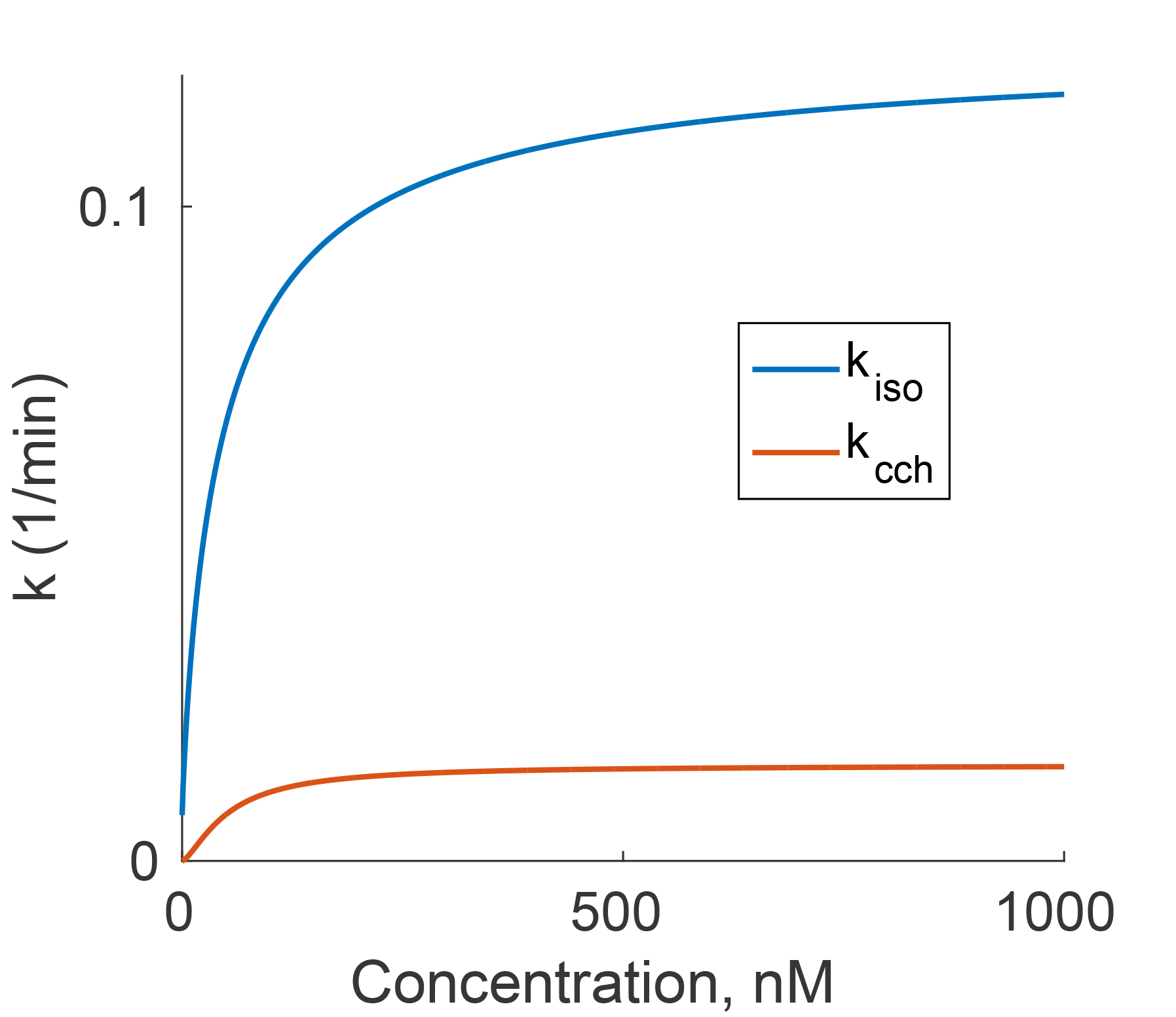
**

**Fig S2:** **Curve fitting for k_iso_ and k_cch_.** Note the faster ignition of k_iso_ for low concentration of ISO versus k_cch_ for low concentration of CCh and that the value of k­_iso_ is an order of magnitude higher than k_cch_ for high concentrations of the drugs.

**
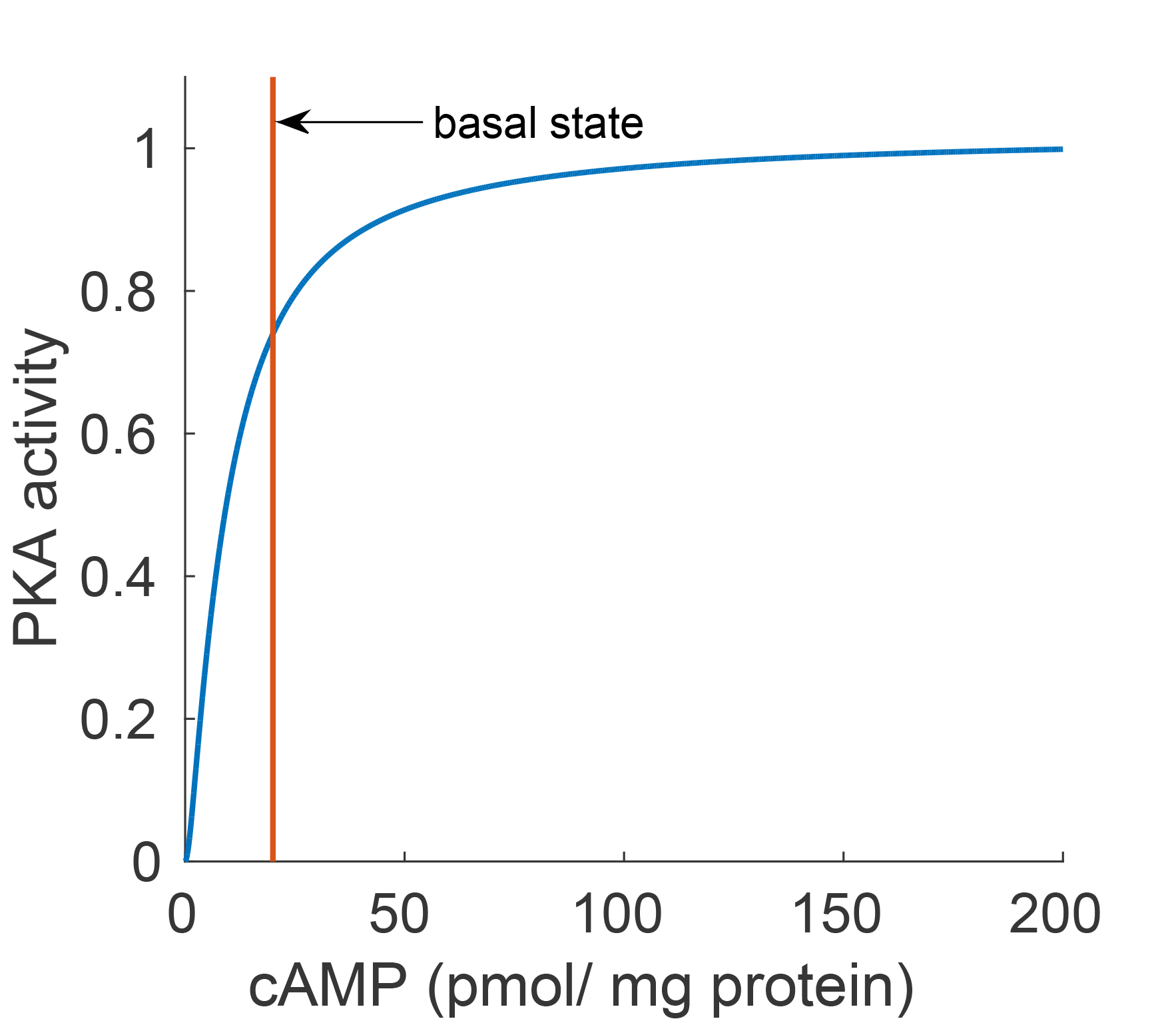
**

**Fig S3:** **cAMP - PKA relationship.** Adapted from the Saucerman *et al.* model (4).

**
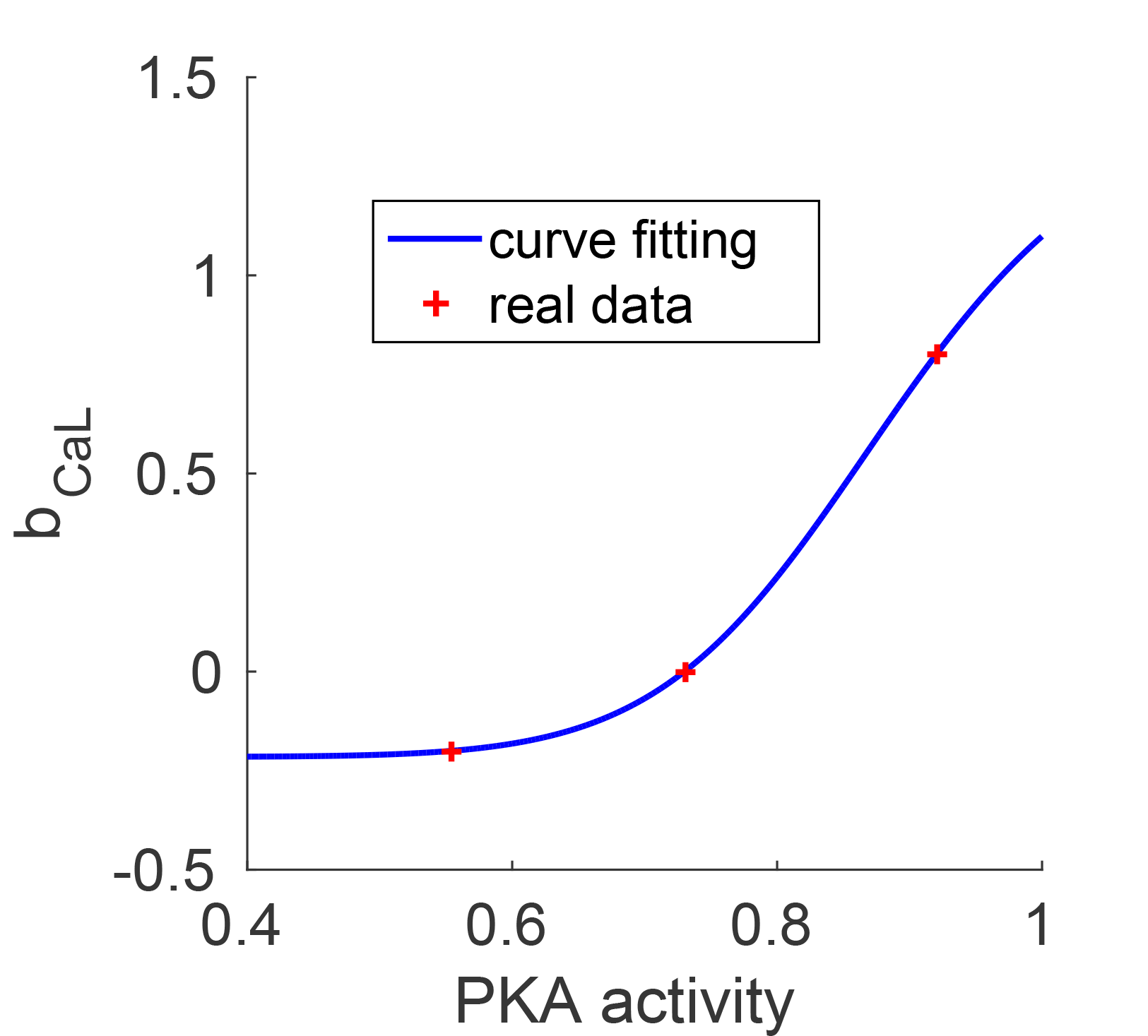
**

**Fig S4: L-type channel modulation by PKA.** Experimental data for curve fitting are taken from (7) and (8).

**
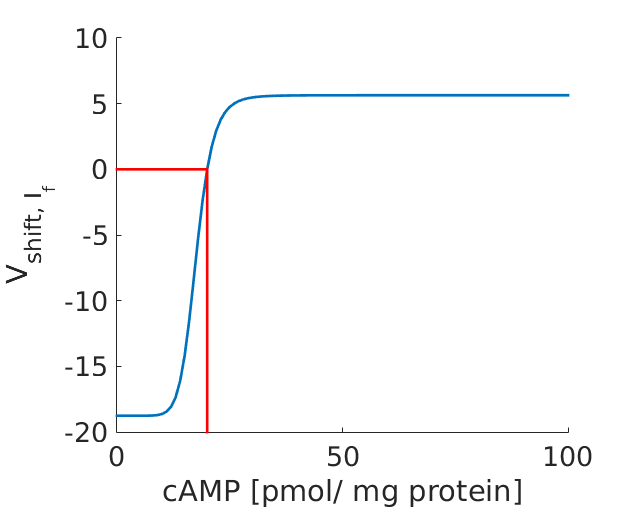
**

**Fig S5:** ***I_f_* shift in the I-V curve as a function of cAMP.** Basal state indicated by the intersection of the two red lines (V_shift_ =0 and [cAMP] = 20 pmol/mg protein).

**
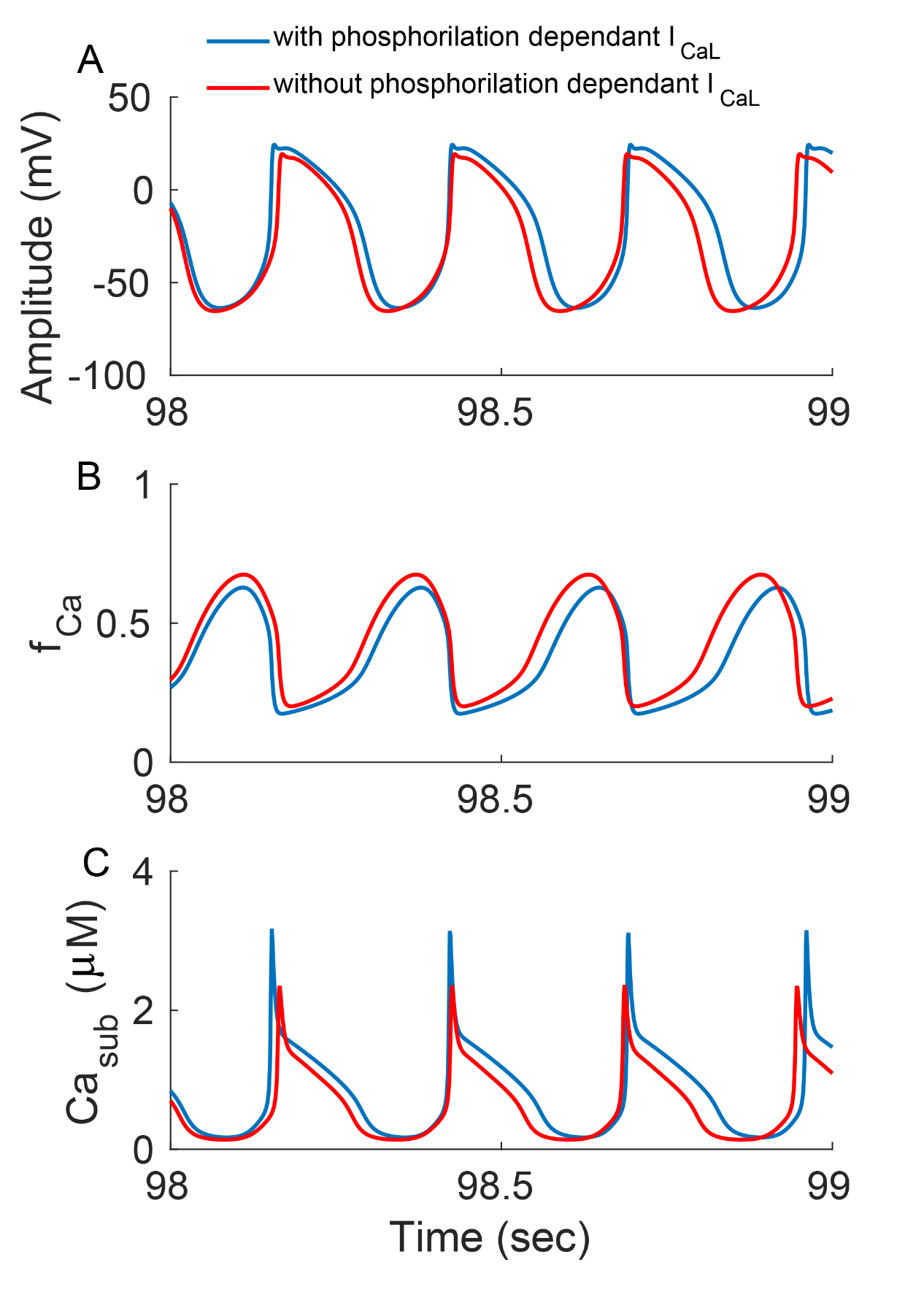
**

**Fig S6: Phosphorylation dependence of *I_CaL_* and its impact on the AP firing rate.**

**
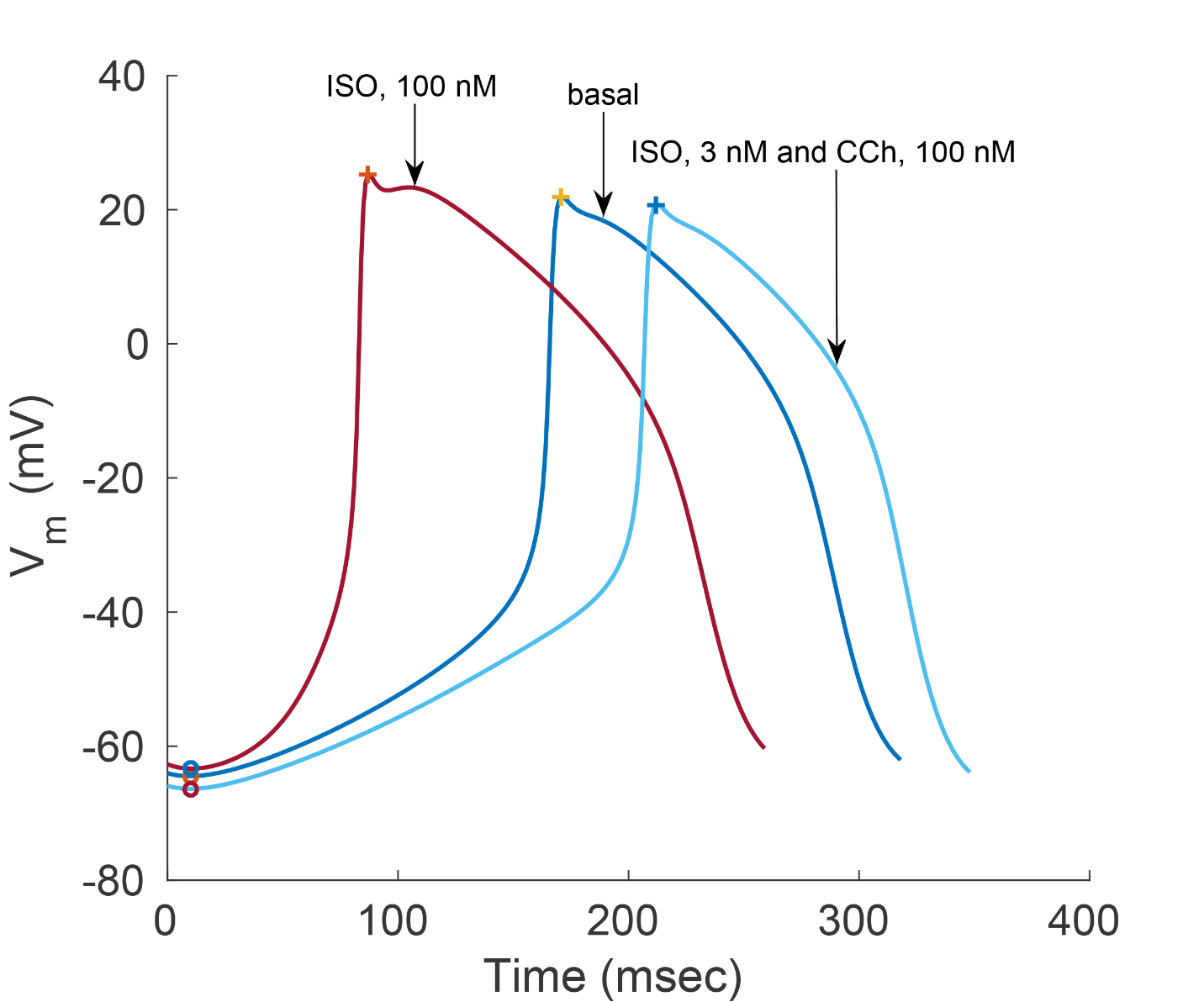
**

**Fig S7: MDP and AP in the presence of ISO or ISO + CCh.** The maximum diastolic potential (MDP) and action potential amplitude (APA) changes under basal conditions, β-AR stimulation (ISO, 100 nM), or vagal stimulation in the presence of a low concentration of β-AR stimulation (ISO, 3 nM and CCh, 100 nM). The changes observed in the MDP and APA are relatively small between the three cases.
